# Beyond the wound: A scoping review of the psychosocial impact of diabetes‐related foot ulcers

**DOI:** 10.1111/dme.70243

**Published:** 2026-02-20

**Authors:** Michelle Hanlon, Brian E. McGuire, Claire MacGilchrist, Ellen Kirwan, Deirdre Ní Neachtain, Ketan Dhatariya, Virginie Blanchette, Hannah Durand, Anda Dragomir, Caroline McIntosh

**Affiliations:** ^1^ School of Psychology & Centre for Pain Research University of Galway Galway Ireland; ^2^ Discipline of Podiatric Medicine, School of Health Sciences University of Galway Galway Ireland; ^3^ Alliance for Research and Innovation in Wounds, College of Medicine, Nursing & Health Sciences University of Galway Galway Ireland; ^4^ Research Services, James Hardiman Library University of Galway Galway Ireland; ^5^ Independent PPI Representative Galway Ireland; ^6^ Elsie Bertram Diabetes Centre, Norfolk and Norwich University Hospitals NHS Foundation Trust Norwich UK; ^7^ Norwich Medical School University of East Anglia Norwich UK; ^8^ Department of Human Kinetics and Podiatric Medicine Université du Québec à Trois‐Rivières Trois‐Rivières Québec Canada; ^9^ Division of Psychology, Faculty of Natural Sciences University of Stirling Scotland UK; ^10^ Montreal Behavioural Medicine Centre Concordia University Montréal Québec Canada

**Keywords:** behaviour change, diabetes‐related foot ulcer, health literacy, person‐centred education, psychosocial factors, self care

## Abstract

**Objectives:**

To explore the emotional consequences of diabetes‐related foot ulcers (DFUs) and examine the psychosocial factors that influence their progression, management and self care behaviours.

**Methods:**

A systematic scoping review was conducted following Arksey and O'Malley's six‐stage framework and the Joanna Briggs Institute guidelines, and reported in accordance with PRISMA‐ScR standards. Studies were eligible if they examined emotional or psychosocial experiences of adults living with DFUs.

**Results:**

Forty‐nine studies were included: 28 cross sectional, 13 qualitative, 5 prospective, 2 randomized controlled trials and 1 case study. Individuals with DFUs experienced heightened emotional distress and substantially reduced health‐related quality of life (HRQOL), largely due to physical limitations, challenges in diabetes self management and fear of future complications. Key psychosocial influences included low self‐efficacy, feelings of powerlessness, loss of independence and perceived burdensomeness. Disparities related to gender, socio‐economic status and cultural background further shaped emotional outcomes and self care behaviours.

**Conclusions:**

Psychosocial factors substantially influence emotional well‐being, treatment adherence and wound healing in people living with DFUs. Integrating psychosocial assessment, tailored education and emotional support into standard care may improve outcomes.

**Practice Implications:**

Routine psychological screening, health literacy–sensitive education and multidisciplinary counselling should be incorporated into DFU management to enhance self care and quality of life.

## Graphical abstract

Living with a DFU is a complex and ongoing journey. From initial onset through active management and long‐term living, individuals can experience a substantial psychosocial burden, including emotional distress, treatment burden and challenges with self‐management. These psychosocial factors may influence engagement with care, treatment adherence and ultimately wound healing and clinical outcomes. This graphical summary illustrates how psychosocial experiences evolve across the DFU journey and highlights key opportunities for intervention. Integrating psychological screening, health literacy–sensitive education and multidisciplinary, person‐centred support into routine care may help reduce psychosocial burden, enhance engagement and improve outcomes for people living with DFUs. Addressing psychosocial needs is therefore essential to delivering holistic, effective DFU care.

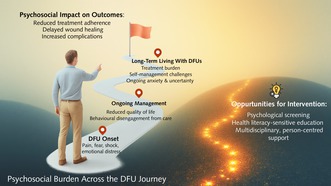

**What's new?**

- Diabetes‐related foot ulcers are associated with substantial psychological and social burden, including high levels of emotional distress, reduced health‐related quality of life and fear of future complications such as amputation.
- Psychosocial factors—including low self‐efficacy, feelings of powerlessness, social isolation and health literacy challenges—significantly influence self‐care behaviours, treatment adherence and wound outcomes.
- Integrating routine psychological screening, tailored education and multidisciplinary psychosocial support into standard DFU care may improve both healing outcomes and patient quality of life.

## 1 INTRODUCTION

Diabetes‐related foot ulcers (DFUs) are among the most prevalent and serious complications of diabetes mellitus (DM) impacting approximately 18.6 million people worldwide each year and leading to considerable morbidity and mortality.^1^, ^2^, ^3^, ^4^ The pathogenesis of DFUs is complex and multifactorial, involving numerous diabetes‐related factors such as uncontrolled hyperglycaemia, increased risk of infection, neuropathy, chronic inflammation and peripheral vascular disease.^5^, ^6^ Additionally, factors such as abnormal biomechanics and inadequate foot care can exacerbate skin breakdown and hinder healing, influencing various stages of ulcer development.^7^ Other risk factors associated with the development of DFUs include a diabetes duration of over 10 years, male gender, older age, the presence of comorbidities and a history of foot ulceration.^8^ At present, the primary objective in managing DFUs is to prevent unnecessary amputations, with most interventions focusing on restoring tissue perfusion and controlling infection.^9^ Current clinical guidelines provide extensive information on preventive and therapeutic strategies designed to reduce the progression of events leading to amputation, including the identification of at‐risk feet, the development of non‐healing wounds and subsequent amputation.^10^ However, the influence of psychosocial factors on the progression and recurrence of ulcers is not yet fully understood, and treatment guidelines for addressing the psychological issues associated with the condition are still in their early stages. Understanding the psychosocial and educational needs of individuals living with DFUs is essential to developing effective person‐centred counselling strategies that promote sustained self care and wound healing.

A number of studies^11^, ^12^, ^13^, ^14^ have shown that individuals living with DFUs frequently experience a range of psychological issues, including stress, anxiety, fear and depression. These psychological factors can exacerbate the physical challenges associated with DFUs, leading to a decline in overall quality of life. Several studies^11^, ^15^, ^16^, ^17^, ^18^ have also shown that psychological distress is linked to a decline in self care behaviours and a lower adherence to treatment regimens, which in turn negatively impacts wound healing and increases the risk of complications and recurrence. However, more extensive research is required to better understand the mechanisms by which psychosocial and behavioural factors may influence foot outcomes so that we can develop more appropriate interventions.^13^, ^19^ This scoping review was thus conducted to examine how emotional and psychosocial factors may influence the management and progression of DFUs, in order to inform the development of a psychological intervention designed to enhance psychosocial functioning and mitigate the progression and recurrence of foot ulcerations.

## 2 MATERIALS AND METHODS

The present scoping review was performed in line with Arksey and O'Malley's six‐stage methodological framework^20^ and the Joanna Briggs Institute guidelines.^21^ It is reported according to the Preferred Reporting Items for Systematic Reviews and Meta‐Analyses extension for Scoping Reviews (PRISMA‐ScR). A protocol for this scoping review was published on HRB Open Research Platform.^22^

### 2.1 Identifying the research question

In accordance with recommendations from the Joanna Briggs Institute,^21^ the PCC (Population, Concept and Context) framework was utilized to formulate the research questions for this scoping review. This framework serves as a guide to facilitate the construction of a clear and meaningful title and the development of subsequent question(s) for a scoping review. For the current review, the authors were interested in individuals with diabetes‐related foot ulcers (P) and exploring how living with this condition impacts their emotional and social well‐being (C) in order to investigate the influence of psychosocial factors may have on DFU healing/ reoccurrence (C).

### 2.2 Participant and public involvement

In line with GRIPP (Guidance for Reporting Involvement of Patients and the Public) recommendations, participant and public involvement was integrated throughout the scoping review process. Input from individuals with relevant experiences was sought to ensure that the research questions and methodology addressed the concerns and perspectives pertinent to this population effectively. This involvement is reported according to the GRIPP checklist (see Table 1 below), which enhances the transparency and relevance of the findings.

**TABLE 1:** GRIPP2 PPI reporting checklist.

Section and topic	Item	Report
1: Aim	Report the aim of PPI in the study	The aim of involving a PPI partner was to enhance the relevance, clarity and practical value of the scoping review by ensuring it addressed issues that mattered to individuals with lived experience of diabetic foot ulcers (DFUs).
2: Methods	Provide a clear description of the methods used for PPI in the study	PPI involvement began with a meeting where I presented the rationale for the review and discussed the proposed research questions. The PPI partner provided feedback on these questions and suggested areas of focus. Upon completion of the review, I invited the partner to review the manuscript and provide input on terminology, the relevance of findings and the accessibility of the language used.
3: Study results	Outcomes—Report the results of PPI in the study, including both positive and negative outcomes	The PPI partner contributed to refining the research questions, highlighting relevant psychological aspects and improving the clarity of the final write‐up. Her feedback helped ensure that the findings were presented in a more accessible and participant‐centred way. A key challenge was the extended time required to recruit a suitable PPI member, which resulted in delays to the project timeline.
4: Discussion and conclusions	Outcomes—Comment on the extent to which PPI influenced the study overall. Describe positive and negative effects	PPI involvement had a meaningful influence on the design, interpretation and presentation of the review. It improved the participant relevance of the research questions and the clarity of dissemination materials. However, recruitment delays highlighted the need for earlier engagement strategies in future studies.
5: Reflections/critical perspective	Comment critically on the study, reflecting on the things that went well and those that did not, so others can learn from this experience	The collaborative engagement with the PPI partner enriched the study by bringing a lived‐experience perspective to both the design and dissemination. Early discussions were particularly helpful in shaping the scope of the review. However, the delay in identifying a suitable PPI partner suggests that integrating PPI at an earlier planning stage is essential to avoid disruption to timelines.

*Note*: Reference: Staniszewska S, Brett J, Simera I, et al. GRIPP2 reporting checklists: Tools to improve reporting of participant and public involvement in research. *BMJ*. 2017;358:j3453. https://doi.org/10.1136/bmj.j3453.

### 2.3 Research questions

1. What are the psychological and emotional consequences of living with diabetes‐related foot ulcers?
2. Do psychosocial factors influence progression or reoccurrence of ulceration in individuals with diabetes‐related foot ulcers?

### 2.4 Eligibility criteria

Studies were included if they focused on adults living with diabetes, specifically addressing diabetes‐related foot ulcers. The eligibility criteria stipulated that these studies must report on the emotional responses and psychosocial impacts experienced by individuals with DFUs. Furthermore, studies that examined the relationship between emotional and psychosocial factors and their influence on self management and wound healing within the context of DFUs were also included.

Studies were excluded if they did not specify the study population, were not published in English or focused on diabetes without addressing the foot. Publications on other diabetes‐related complications, other chronic conditions or emotional and psychosocial impacts unrelated to diabetes‐related foot issues were also excluded. Additionally, studies on self management and wound healing that did not specifically examine diabetes‐related foot ulcers were excluded. Finally, general literature reviews were excluded to ensure that conclusions are based on original research rather than interpretations or summaries of previous work, and studies were combined if they utilized the same group of participants.

### 2.5 Justification for inclusion and exclusion criteria

This inclusion and exclusion criterion ensured a targeted exploration of the unique emotional challenges and psychosocial impacts faced by individuals living with DFUs, maintaining the specificity of the research. By excluding broader diabetes‐related emotional and psychosocial issues, the study avoids potential dilution of findings, recognizing that emotional and psychosocial experiences can vary significantly across different diabetes complications. Focusing solely on DFUs allows relevant and actionable insights, which are crucial for developing targeted interventions that address both the emotional and social dimensions of living with these ulcers.

Table 2 below presents the inclusion and exclusion criteria for the scoping review, structured according to the PCC framework.

**TABLE 2:** Inclusion and exclusion criteria with justifications.

PCC framework	Inclusion criteria	Exclusion criteria	Justification
Population (Individuals with DFU)	Publications that report on studies related to DFUs.	Any publication about diabetes that does not relate to the foot. Any publication relating to other diabetes‐related lower extremity complications (diabetes‐related foot infections) or ulcers.	The objective of the study is to investigate DFUs specifically. Including publications that address general diabetes management or unrelated diabetic complications could dilute the focus and relevance of the findings, making it challenging to draw specific conclusions about DFUs.
Concept (Emotional implications and Psychosocial impact)	Publications that report on emotional responses experienced as a result of living with DFU. Publications that report on psychosocial impacts experienced as a result of living with DFU.	Any publication that explores the emotional implications of living with diabetes but does not explore diabetes‐related foot specifically. Any publication relating to emotional implications of other chronic conditions. Any publication that explores the psychosocial impacts of living with diabetes as a condition but does not look at the diabetic foot. Any publication relating to the psychosocial impact of living with other chronic conditions.	This inclusion criteria ensures a targeted exploration of the specific emotional challenges and psychosocial impact faced by individuals living with DFUs. This exclusion criteria maintains the specificity of the research, preventing dilution of findings with broader diabetes‐related emotional and psychosocial issues that may not apply to DFUs. Emotional and psychosocial experiences can vary widely among different complications of diabetes, and focusing solely on DFUs ensures relevant and actionable insights. Understanding these impacts is essential for developing targeted interventions that address both the emotional and social dimensions of living with DFUs.
Context (Management, wound healing and recurrence)	Publications that report on emotional and psychosocial factors and their impact on self management and wound healing in DFU.	Any publication that looks at the role that emotional and psychosocial factors have on self management and wound healing but does not explore this in relation to the diabetic foot specifically. Any publications relating to self management and wound healing in other chronic conditions.	This inclusion criteria focuses on understanding how emotional and psychosocial factors specifically influence self management and wound healing in those with DFUs. By examining these factors, the research can uncover insights that inform holistic treatment approaches, addressing both physical healing and the emotional well‐being of people living with DFUs.

Abbreviation: DFU, diabetes‐related foot ulcer.

### 2.6 Search strategy and study selection process

**FIGURE 1:**
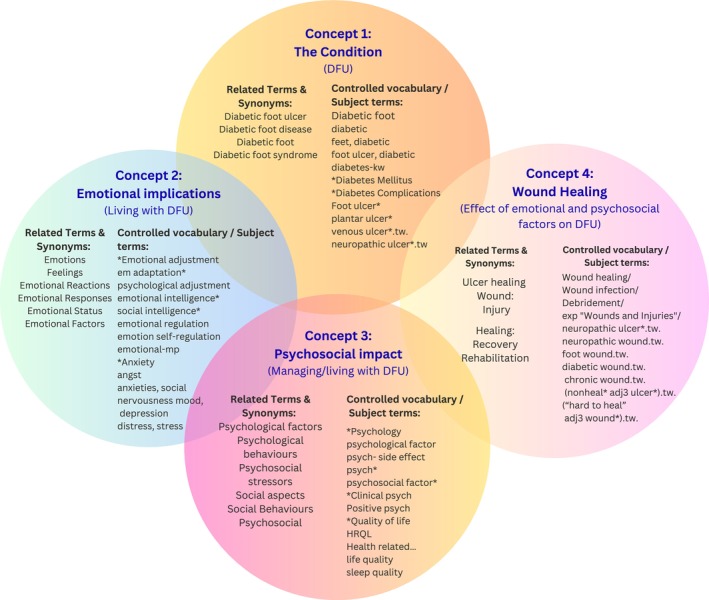
Visual representation of search terms used in the scoping review.

A three‐step search strategy was employed for this study. First, an initial search of two databases, PubMed and PsycINFO, was undertaken, followed by an analysis of the text words contained in the title and abstract of retrieved papers. Utilizing the PCC framework, ideas were then expanded on, using search terms and appropriate thesaurus terms and synonyms. Subsequently, a comprehensive search was undertaken across OVID (Medline), EMBASE, CINAHL, PsycINFO, SCOPUS, the Cochrane Library and Web of Science Core Collection, incorporating all identified keywords and index terms. Finally, grey literature was sought through the ProQuest E‐Thesis Portal and Lenus, along with a review of the reference lists of all included articles for additional sources. Support was enlisted from an academic librarian at the University of Galway to access a number of texts that were found via the ProQuest E‐Thesis Portal and Lenus, and inter‐library loans were secured. Figure 1 below provides a visual representation of the search terms utilized in this study. Additionally, a table is included in File S1 for further reference.

### 2.7 Data extraction

#### 2.7.1 Study selection

All identified citations were imported to the Covidence software, where duplicates were removed. All citations were double screened independently by two authors, MH and EK, first by title and abstract and then by full text. The citations were assessed based on eligibility and exclusion criteria. Any disagreements between the authors were discussed and consensus was reached.

#### 2.7.2 Data extraction and synthesis

A data‐charting form was developed in Microsoft Excel by the lead author (MH) and approved by the research team. The form was piloted on three full‐text articles for each category by two reviewers (MH and EK). The data was then extracted from all included articles by one reviewer (MH), and it was then reviewed and verified by a second member of the research team (EK).

## 3 RESULTS

Results are presented in line with the PRISMA‐ScR. The characteristics of all included studies are summarized below in Figures 3 and 4 and in detail in the table provided in File S2.

### 3.1 Study selection and characteristics

#### 3.1.1 Study selection

**FIGURE 2:**
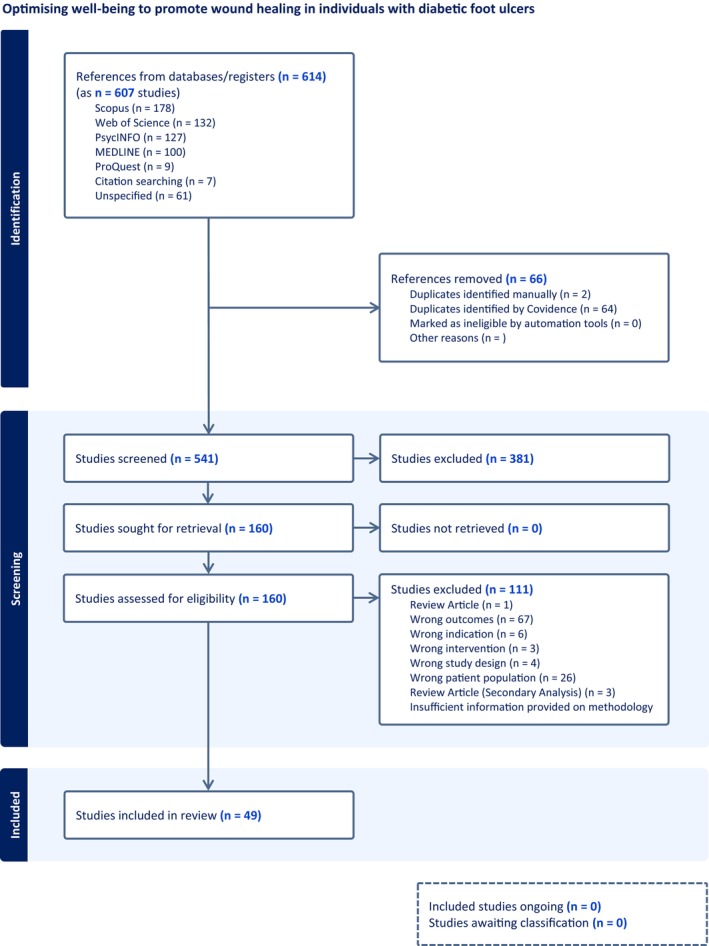
Prisma flow diagram.

This scoping literature review initially identified 614 articles through electronic database searches (see Figure 1 for search strategy). After removing duplicates, 544 articles were assessed for relevance. Following the screening of titles and abstracts, 381 studies deemed irrelevant were excluded. The remaining full‐text articles were then evaluated for eligibility, leading to the exclusion of 111 articles that did not meet the inclusion criteria. The reasons for exclusion included: incorrect population, inappropriate outcomes or indications, inappropriate study design/review article, duplicate publications of study and insufficient information. Ultimately, 49 studies met the inclusion criteria and were included in the final review (see PRISMA flow chart in Figure 2 below).

#### 3.1.2 Study characteristics

**FIGURE 3:**
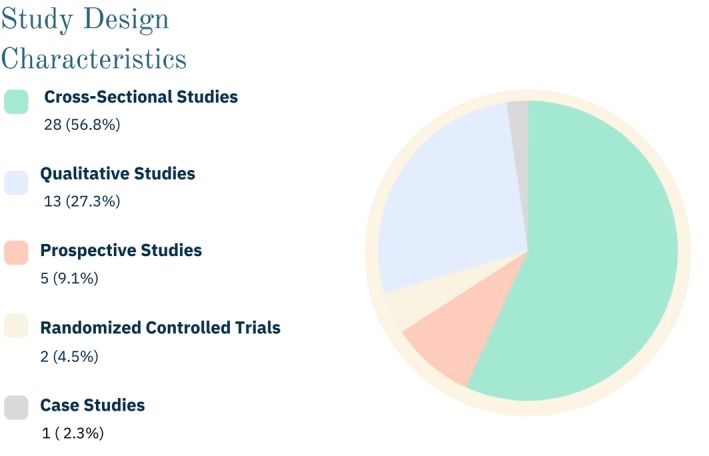
Overview of study design characteristics.

**FIGURE 4:**
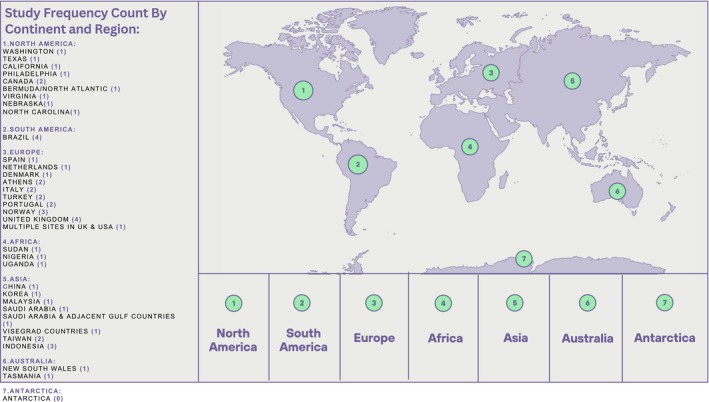
Study frequency count by continent and region.

Out of the 49 studies included in this review, 28 were cross sectional, 13 were qualitative, 5 were prospective, 2 were randomized controlled trials and 1 was a case study report. A significant number of these studies were conducted in European countries (19), primarily in clinical settings such as diabetes centres and wound care clinics. A detailed breakdown of study types, the countries they were conducted in and the study settings is provided in the table in File S2 and an overview is provided in Figures 3 and 4. In total, there were 71, 632 participants across all studies. However, it should be noted that one large study^9^ included 65,126 persons, of whom 63,632 were participants without DFUs. One study^23^ noted an 18‐year‐old participant, while another study^24^ reported a participant who was 98 years old. However, the average age of participants across all studies was approximately 64.36 years. Two studies did not provide a breakdown by gender but in the 49 studies that were included 56.8% of participants were men and 43.2% were women. The earliest included study was from 2000; however, 75% of the articles were published after 2010. table 1 in File S2 presents all studies that met the inclusion criteria discussed in this scoping review.

### 3.2 Key findings

**FIGURE 5:**
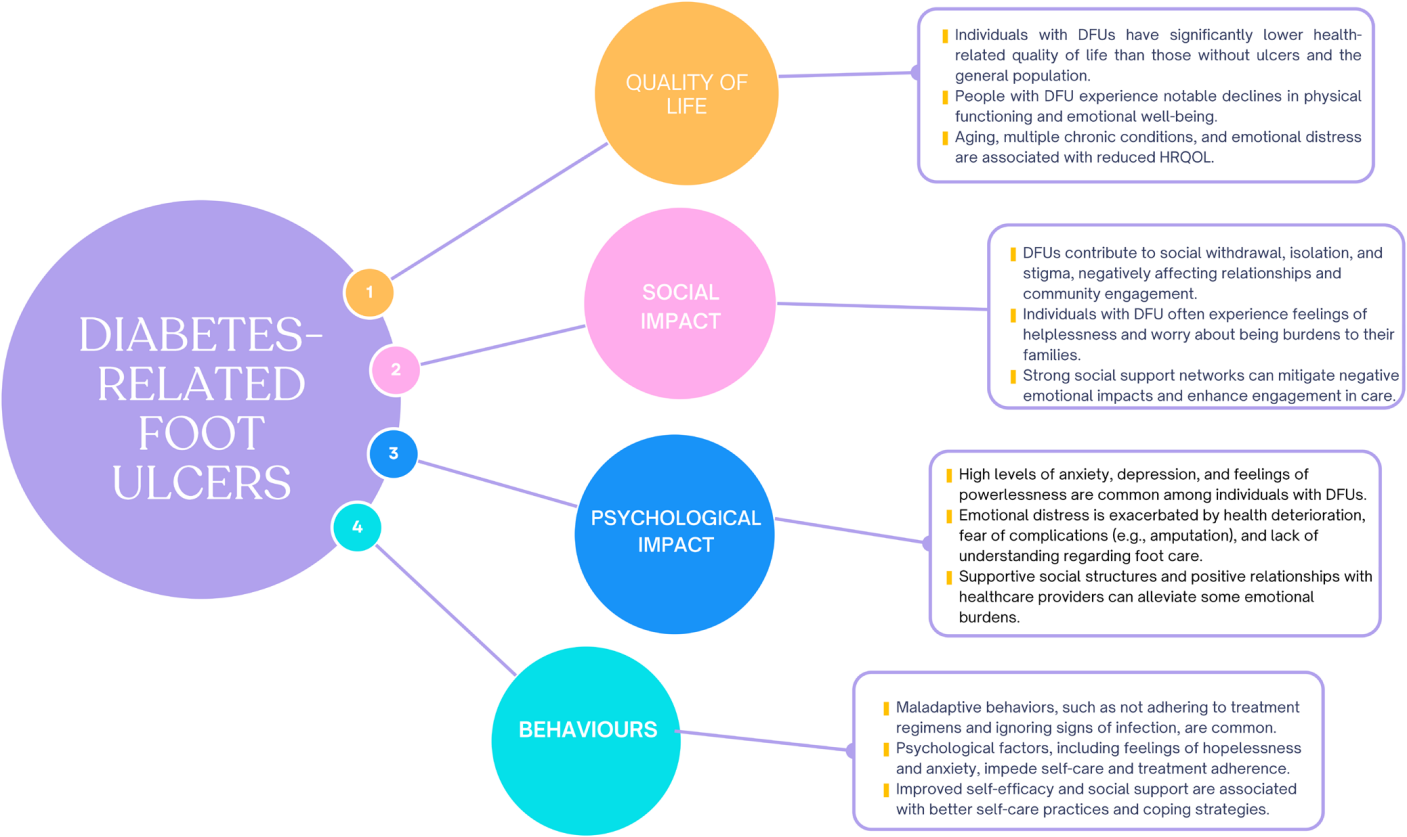
Summary of key findings.

A summary of key findings is illustrated in Figure 5, with a detailed analysis presented in the following section.

#### 3.2.1 Psychological and emotional consequences of DFUs

Across studies, individuals with DFUs consistently reported significantly lower HRQOL compared to those with diabetes without ulcers and the general population.^24^, ^25^, ^26^, ^27^, ^28^, ^29^, ^30^, ^31^, ^32^, ^33^, ^34^, ^35^, ^36^, ^37^, ^38^, ^39^, ^40^, ^41^, ^42^, ^43^, ^44^, ^45^, ^46^, ^47^, ^48^, ^49^, ^50^, ^51^, ^52^, ^53^, ^54^, ^55^, ^56^, ^57^, ^58^, ^59^, ^60^, ^61^, ^62^, ^63^ Emotional distress was commonly expressed as heightened anxiety, depression and feelings of powerlessness. These emotions were often rooted in deteriorating health, the ongoing burden of managing diabetes and DFUs, fear of future complications (particularly amputation), physical limitations, loss of independence and the perception of being a burden on family members.^23^, ^33^, ^35^, ^43^, ^45^, ^62^, ^64^, ^65^ Additionally, a lack of understanding about foot care and diabetes management was reported to amplify fear and guilt, further exacerbating emotional distress. Individuals also described challenges in maintaining daily foot inspections, consistently using prescribed footwear and following clinical advice, with emotional distress commonly contributing to disengagement. The duration of diabetes, the presence of complications and a history of amputation or fear of amputation were also strongly linked to worsening mental health outcomes over time.^25^, ^26^, ^27^, ^30^, ^41^, ^45^

#### 3.2.2 Psychosocial impact and behaviours: Ulcer progression and recurrence

This review also highlighted the significant psychosocial challenges associated with DFUs, underscoring their profound impact on relationships, social engagement and overall quality of life. Many studies reported that participants frequently experienced feelings of helplessness, fear, worry and low mood and that this often led to social withdrawal and isolation.^15^, ^16^, ^24^, ^29^, ^30^, ^37^, ^39^, ^40^, ^41^, ^48^, ^49^, ^51^, ^59^, ^60^, ^62^, ^64^, ^66^ Social stigma and the perception of being a burden to family members further contributed to reduced social interactions and disengagement from community activities. Gender disparities were notable, with several studies reporting that women tend to experience poorer mental health outcomes.^40^, ^56^, ^57^ Other factors shown to negatively affect HRQOL and self care compliance included lower educational attainment, lower socio‐economic status, pain, older age, multimorbidity and perceived physical limitations and decreased self‐efficacy.^12^, ^23^, ^31^, ^32^, ^33^, ^35^, ^36^, ^40^, ^42^, ^43^, ^49^, ^50^, ^52^, ^53^, ^55^, ^62^, ^67^ In relation to health behaviours, several studies identified maladaptive patterns that adversely impacted healing and outcomes.^32^, ^43^, ^64^ These included not reducing activities to give the ulcer time to heal, failure to wear prescribed footwear and ignoring pain or early signs of infection. Additionally, a number of studies reported that many individuals did not consistently engage in daily examinations or adhere to treatment regimens as a result of feelings of hopelessness, powerlessness, anxiety and depression.^15^, ^25^, ^27^, ^34^, ^39^, ^48^, ^49^, ^62^, ^64^

## 4 DISCUSSION

This review examined the emotional and psychosocial consequences of diabetes‐related foot ulcers (DFUs) and the potential influence of these factors on ulcer progression and recurrence. Across the included studies, anxiety, depression and emotional distress were frequently reported among individuals living with DFUs.^24^, ^25^, ^26^, ^27^, ^28^, ^29^, ^30^, ^31^, ^32^, ^33^, ^34^, ^35^, ^36^, ^37^, ^38^, ^39^, ^40^, ^41^, ^42^, ^43^, ^44^, ^45^, ^46^, ^47^, ^48^, ^49^, ^50^, ^51^, ^52^, ^53^, ^54^, ^55^, ^56^, ^57^, ^58^, ^59^, ^60^, ^61^, ^62^, ^63^ While such responses may be anticipated in the context of a chronic and potentially debilitating complication, the reviewed literature emphasizes the extent to which psychological well‐being influences self care engagement, health behaviours and diabetes management more broadly.^15^, ^16^, ^25^, ^27^, ^34^, ^39^, ^48^, ^49^, ^62^, ^64^ In parallel, DFUs were repeatedly associated with declines in health‐related quality of life (HRQOL), particularly in domains related to physical function and emotional well‐being. The magnitude of these impacts appeared to vary across sociodemographic groups; women were more likely to report poorer psychological outcomes than men, and additional risk factors for reduced HRQOL included older age, lower educational attainment, socio‐economic disadvantage and the presence of multiple comorbidities.^12^, ^23^, ^31^, ^32^, ^33^, ^35^, ^36^, ^40^, ^42^, ^43^, ^49^, ^50^, ^52^, ^53^, ^55^, ^56^, ^57^, ^62^, ^67^ In addition, several studies identified practical and psychological barriers to effective self management. Limited foot care knowledge, low health literacy and negative illness perceptions were commonly linked to reduced engagement in preventive behaviours and poorer treatment adherence.^23^, ^32^, ^33^, ^52^, ^68^ Collectively, these findings highlight the need for person‐centred care approaches that address not only the psychosocial burden of DFUs but also the broader social contexts in which individuals manage their condition.

Importantly, several psychosocial constructs emerged as moderators of individuals' experiences with DFUs. Psychological resilience and self‐efficacy were associated with better emotional adjustment and stronger adherence to recommended care routines.^39^, ^45^, ^68^ Several studies also indicated that supportive social structures—such as strong family and community ties and positive relationships with healthcare providers—helped mitigate negative emotional effects and led to improved HRQOL and more consistent engagement in foot care.^16^, ^25^, ^43^, ^44^, ^49^, ^55^, ^63^ These patterns are consistent with established psychosocial and behavioural health models, which emphasize the interplay between personal coping resources and social environments in shaping health outcomes. Taken together, the findings reinforce that the burden of DFUs extends well beyond their physical manifestations, underscoring the importance of management strategies that incorporate psychosocial dimensions. Interventions that foster emotional well‐being, strengthen support networks and preserve functional independence are thus very important to enhancing quality of life for this population.

However, although awareness of the psychosocial dimensions of DFUs is increasing, the current evidence base remains limited. Most studies included in this review employed cross sectional or observational designs, restricting the ability to infer causality. Few investigations have directly explored the mechanisms through which psychosocial variables influence ulcer healing or recurrence or evaluated the effectiveness of targeted psychosocial interventions. Future research employing longitudinal and interventional methodologies is essential to clarify these relationships.

### 4.1 Education perspective

The findings of this review emphasize that psychosocial distress, low self‐efficacy and limited health literacy can substantially hinder effective self care among individuals with diabetes‐related foot ulcers. These insights highlight the critical role of education and counseling in DFU management. Educational strategies should move beyond information provision to actively foster coping skills, confidence and emotional resilience. Tailored education—sensitive to literacy level, cultural background and individual readiness for behavior change—can help people living with DFUs to better understand the relationship between emotional well‐being and wound healing. Incorporating structured counselling and peer or family support within multidisciplinary care may strengthen engagement, reduce recurrence and improve overall quality of life.

## 5 CONCLUSION

Diabetes‐related foot ulcers are a serious and globally prevalent complication of diabetes mellitus, associated with substantial physical, psychological and social burdens. This review highlights the multifaceted impact of DFUs on health‐related quality of life and reinforces the need for more integrated, person‐centred approaches to care. Effective management must extend beyond wound treatment to include holistic strategies that address psychological well‐being, enhance education and strengthen social support systems. Key priorities include routine psychological screening, accessible and comprehensive foot care education, regular foot assessments and multidisciplinary care tailored to individual risk profiles. Interventions that support emotional resilience and self‐efficacy, while fostering consistent self care and treatment adherence, may lead to improved clinical and psychosocial outcomes. Addressing disparities related to gender, socio‐economic status and cultural context is also essential to delivering equitable and inclusive care. While current evidence suggests psychosocial factors may influence the progression and recurrence of DFUs, causal mechanisms remain poorly understood. Further longitudinal and interventional research is needed to clarify these relationships and inform the development of targeted, evidence‐based interventions. Advancing this area of inquiry is critical to improving both clinical outcomes and quality of life for individuals living with DFUs.

## 6 STRENGTHS AND LIMITATIONS

This review, part of a PhD project aimed at developing a psychological intervention for DFUs, employed a scoping review methodology due to several advantages. First, the exploratory nature of this approach is beneficial, as the relationship between psychological factors and DFUs remains underexplored. A scoping review facilitates a broad examination of relevant aspects such as depression, anxiety, stigma and coping strategies, helping to map existing evidence and identify research gaps. Additionally, this methodology systematically highlights gaps in the literature, guiding future research directions and focusing on impactful psychological factors. Finally, the flexibility of scoping reviews, which do not impose strict inclusion/exclusion criteria, allows for a more adaptable approach in a field characterized by varied methodologies.

The review employed a thorough three‐step literature search across multiple databases and grey literature sources, ensuring a wide exploration of studies related to the emotional and psychosocial impacts of DFUs. This comprehensive approach included diverse study designs—cross sectional, qualitative and randomized controlled trials—enhancing the richness of the findings. Using the PCC framework, clear research questions targeted the psychological and emotional consequences of living with DFUs, revealing interconnected themes regarding health‐related quality of life. The findings underscore the complex interplay between physical health, emotional well‐being and social support, offering insights for developing targeted psychological interventions to improve psychosocial functioning and reduce DFU progression.

However, several limitations must be acknowledged. Many studies did not adjust for confounding variables such as income, healthcare access and comorbid conditions like obesity and renal disease, potentially affecting the interpretation of associations between psychosocial factors and DFU outcomes. The predominance of cross sectional designs introduces risks of response bias and limits causal inferences, restricting generalizability. Furthermore, the exclusion of publications not published in English may have resulted in the omission of relevant evidence and introduced language bias. Despite these limitations, this review offers a comprehensive synthesis of the emotional and psychosocial dimensions of living with DFUs and identifies crucial areas for future intervention and research.

In conclusion, this review demonstrates that emotional distress, psychosocial strain and diminished self‐efficacy play a central role in shaping how people living with DFUs manage their condition and engage in self care. These factors influence not only daily foot care practices but also broader health behaviours that affect healing and the risk of recurrence. Collectively, the findings reinforce the need for DFU care to extend beyond physical wound management and to systematically incorporate psychosocial assessment, emotional support and person‐centred education. Embedding these components into routine clinical practice may strengthen self management, improve treatment adherence and ultimately enhance both healing outcomes and quality of life for those living with DFUs.

## AUTHOR CONTRIBUTIONS

**Michelle Hanlon:** Conceptualization, Formal Analysis, Investigation, Methodology, Project Administration, Validation, Visualization, Writing—Original Draft Preparation, Writing—Review & Editing. **Brian E. McGuire:** Conceptualization, Funding Acquisition, Investigation, Methodology, Supervision, Validation, Visualization, Writing—Review & Editing. **Claire MacGilchrist:** Conceptualization, Investigation, Methodology, Supervision, Validation, Visualization, Writing—Review & Editing. **Ellen Kirwan:** Methodology, Investigation, Validation, Writing—Review. Deirdre Ní Neachtain: Validation, Visualization, Writing—Review. **Ketan Dhatariya:** Methodology, Validation, Visualization, Writing—Review. **Virginie Blanchette:** Methodology, Validation, Visualization, Writing—Review. Hannah Durand: Methodology, Validation, Writing—Review. **Anda Dragomir:** Methodology, Validation, Writing—Review. **Caroline McIntosh:** Conceptualization, Funding Acquisition, Investigation, Methodology, Supervision, Validation, Visualization, Writing—Review & Editing.

## FUNDING INFORMATION

This work was supported by a grant from the Health Research Board (HRB) Ireland (CDA‐PA‐2019‐011). The funders had no role in study design, data collection and analysis, interpretation, decision to publish or preparation of the manuscript.

## CONFLICT OF INTEREST STATEMENT

The authors declare that they have no known competing financial interests or personal relationships that could have appeared to influence the work reported in this paper.

## Supporting information


Data S1:

Data S2:
