# Supplementary material for: Beyond the wound: A scoping review of the psychosocial impact of diabetes‐related foot ulcers
Source: Diabet Med. 2026 Feb 20;43(4):e70243. doi: 10.1111/dme.70243 (PMC12982655; doi:10.1111/dme.70243)
Supplement: Supplementary file 1 — Data S1: [file DME-43-e70243-s002.docx]

**Supplementary Data: Search Strategy**

|  | **Concept 1** | **Concept 2** | **Concept 3** | **Concept 4** |
| --- | --- | --- | --- | --- |
| **Key concepts** | **The Condition**  (Diabetic Foot Ulcer) | **Emotional implications**  (Living with Diabetic Foot Ulcer) | **Psychosocial impact**  (Managing/living with Diabetic Foot Ulcer) | **Wound Healing**  (Effect emotional and psychosocial factors- & associated behaviours-have on wound healing in Diabetic Foot Ulcer) |
| **Free text terms / natural language terms** | Related Terms & Synonyms:  Diabetic foot ulcer  Diabetic foot disease  Diabetic foot  Diabetic foot syndrome | Related Terms & Synonyms:  Emotions  Feelings  Emotional Reactions  Emotional Responses  Emotional Status  Emotional Factors | Related Terms & Synonyms:  Psychological factors  Psychological behaviours  Psychosocial stressors  Social aspects  Social Behaviours  Psychosocial | Related Terms & Synonyms:  Ulcer healing  Wound:  Injury  Healing:  Recovery  Rehabilitation |
| **Controlled vocabulary terms / Subject terms**  (MeSH terms, Emtree terms) | Diabetic foot  diabetic foot  feet, diabetic  foot, diabetic  foot ulcer, diabetic  diabetes-kw  *Diabetes Mellitus  *Diabetes Complications  Foot ulcer*  plantar ulcer*  venous ulcer*.tw.  neuropathic ulcer*.tw | *Emotional adjustment  em adaptation*  psychological adjustment  emotional intelligence*  social intelligence*  emotional regulation  emotion self-regulation  emotional-mp  *Anxiety  angst  anxieties, social  nervousness mood, depression distress, stress | *Psychology  psychological factor  psych- side effect  psych*  psychosocial factor*  *Clinical psych  Positive psych  *Quality of life  HRQL  Health related…  life quality  sleep quality | Wound healing/  Wound infection/  Debridement/  exp "Wounds and Injuries"/ neuropathic ulcer*.tw.  neuropathic wound.tw.  foot wound.tw.  diabetic wound.tw.  chronic wound.tw.  (nonheal* adj3 ulcer*).tw.  (“hard to heal” adj3 wound*).tw. |
