# Supplementary material for: Beyond the wound: A scoping review of the psychosocial impact of diabetes‐related foot ulcers
Source: Diabet Med. 2026 Feb 20;43(4):e70243. doi: 10.1111/dme.70243 (PMC12982655; doi:10.1111/dme.70243)
Supplement: Supplementary file 2 — Data S2: [file DME-43-e70243-s001.docx]

**Characteristics of Included Studies**

|  | **Reference/Country** | **Research Design** | **Population/ Sample Size** | **Measures used** | **Aim** | **Results overview** | **Psychological/ emotional consequences of living with DFU** | **Psychosocial impact and behaviours** | **Do psychosocial factors influence progression or reoccurrence of ulceration** |
| --- | --- | --- | --- | --- | --- | --- | --- | --- | --- |
| 1. | (Abdelgadir et al. 2009)  Sudan | Cross-sectional study (Questionnaires) | 120  N with DFU=60 | **The Medical Outcomes Study questionnaire** (Stewart 1992); **Antonovsky’s Sense of Coherence scale (SOC-13)** (Antonovsky 1993); **Gothenburg Quality of Life Instrument (GQL)** (Tibblin et al. 1990). | To investigate the influence of lower limb amputation on health-related quality of life (HRQOL) in Sudanese diabetic subjects. | Sudanese individuals with diabetes who have undergone lower limb amputation (LLA) face poor health-related quality of life (HRQOL). Key factors negatively impacting HRQOL include the duration of diabetes, symptoms, and time since amputation, while a higher sense of coherence is linked to better emotional and social well-being. | This study highlights that individuals with DFUs experience significantly lower health-related quality of life (HRQOL) compared to those without such complications. The findings indicate that longer durations of diabetes and amputation correlate with poorer HRQOL, emphasizing the emotional distress associated with physical limitations and mobility challenges. Participants reported feelings of fatigue, depression, and anxiety, which were exacerbated by the presence of symptoms related to diabetes complications. Additionally, the study notes a connection between a low sense of coherence—reflecting an individual’s capacity to manage stress and maintain psychological well-being—and reduced HRQOL. Despite these challenges, the presence of supportive family relationships contributed positively to emotional well-being, suggesting that social structures can mitigate some of the negative impacts of DFUs. Overall, the study underscores the multifaceted emotional burden faced by diabetic individuals with foot complications and the need for comprehensive support systems to address both physical and psychological health. | The duration of diabetes and time since amputation are linked to worsening mental health outcomes, indicating that longer illness duration exacerbates psychosocial challenges. Additionally, the study highlights the importance of social support, as strong family ties were associated with improved emotional well-being. However, many patients exhibited behaviors such as non-compliance with foot care and a reluctance to seek medical help, stemming from feelings of hopelessness and inadequate diabetes education. This underscores the need for targeted interventions that address both the physical complications of diabetes and the emotional and social factors influencing patient behavior and health outcomes.  Key factors negatively impacting HRQOL included the duration of diabetes, symptoms, and the time since amputation, while a higher sense of coherence was linked to better emotional and social well-being. | This paper focuses on the influence of lower limb amputation on health-related quality of life (HRQOL) in Sudanese diabetic subjects.  However, the paper does suggest that psychosocial factors do influence the progression and recurrence of ulceration in diabetic patients. It highlights that emotional distress, feelings of hopelessness, and inadequate diabetes education negatively impact patients' adherence to foot care, which can lead to a higher risk of complications, including ulceration. Additionally, the study indicates that longer durations of diabetes and associated emotional challenges are linked to poorer health outcomes, reinforcing the role of psychosocial elements in the management and recurrence of diabetic foot ulcers. |
| 2. | (Ahroni & Boyko 2000)  Washington, USA | Observational, prospective study (interview, examination and questionnaires) | 331  N with DFU=  Time 1: 47 (14.2%)  Time 2: 87 (26.3%) | Short-Form 36-Item Survey (SF-36) | To examine the responsiveness of the Medical Outcomes Study 36-Item Short Form (SF-36) to the development of diabetes complications over time. | A study of elderly male veterans with long-term diabetes shows increased comorbidities over time, as reflected in declines in six SF-36 health scales related to worsening complications. Despite small effect sizes, these scales were responsive to changes in health status, supporting their use in longitudinal research. | In a cohort of elderly male veterans with long-standing diabetes, a high level of comorbidity was observed, which generally worsened over time. Those in the longer follow-up tertile experienced a significantly greater decline in the Mental Health (MH) scale of the SF-36 compared to those in the shorter follow-up tertile. On two scales (Mental Health and Emotional Role/RE), most of the subjects had a T2 score within five points of their baseline score T1. Subjects in the longer follow-up tertile had significantly more decline in the MH scale of the SF-36 than subjects in the shorter follow-up tertile.  Findings indicated that subjects experienced a notable decline in physical functioning, general health, and vitality over time, particularly among those with worsening diabetes complications. While some participants reported stable or improved health, a substantial number experienced increased diabetes-related complications, which correlated with lower scores in several SF-36 domains. Interestingly, the mental health scale showed minimal change, suggesting that despite a decline in physical health, some individuals may maintain a stable mental outlook. However, the overall trend highlights that the presence of DFUs and related complications can lead to decreased quality of life, increased emotional distress, and a sense of worsening health perception over time. | With regard to the SF-36 on six of the scales (GH, PF, SF, RP, BP, and VT), most of the subjects had a score lower by more than five points at T2.Subjects in the medium follow-up tertile had greater declines in five scales of the SF-36 (GH, PF, SF, RP, and VT) than those with shorter follow-up.  Many participants reported a decline in quality of life, as reflected in lower scores on the SF-36 health survey, particularly in domains related to physical functioning and general health. The presence of diabetes-related complications, such as neuropathy and hypertension, was commonly associated with increased emotional distress and a perception of worsening health. Participants displayed behaviors characterized by reluctance to seek help, often internalizing their struggles and feelings of helplessness. While some individuals showed stability or improvement in certain health aspects, the overall trend indicated that many experienced deterioration in their physical and mental health, emphasizing the need for comprehensive support systems that address both the physical and psychological challenges faced by those living with DFUs. | The text indicates that psychosocial factors may influence the progression or recurrence of ulceration in individuals with diabetes. It reveals a significant association between the development of diabetes-related complications—specifically renal and neuropathic issues—and declines in health status as measured by the SF-36 scales. The analysis shows that patients with worsening complications exhibited greater declines in physical functioning and general health scores compared to those who improved or remained stable. Although the study notes that the effect of psychosocial factors was not explicitly measured, the observed correlations suggest that these factors could contribute to how individuals experience and report their health status as their diabetes complications progress. The importance of using disease-specific measures, like the DCC, alongside generic health measures is emphasized, as they may better capture the nuances of diabetes-related psychosocial impacts and their relationship to complications like ulceration. |
| 3. | (Al Ayed et al. (2020).  Saudi Arabia | Cross-sectional study (Questionnaire) | 81  N with DFU= 81 | Short-Form 36-Item Survey (SF-36) | To estimate the HRQOL and its related risk factors in patients with foot ulcers associated with type 2 diabetes mellitus (T2DM). | Individuals with DFU had significantly lower health-related quality of life (HRQOL) across all SF-36 domains. | The study highlights the significant emotional and mental health impact of diabetic foot ulcers (DFUs). Patients with DFUs reported markedly lower health-related quality of life (HRQOL) across various domains, particularly in physical functioning and emotional well-being. The presence of DFUs was associated with increased feelings of pain, anxiety, and depression, which were exacerbated by factors such as gender, age, and the number of diabetes-related complications. Notably, women experienced poorer HRQOL compared to men, reflecting potential societal and healthcare system barriers that affect their access to care and support.  The study reports that the part played by emotions is influenced by the level of education, occupation, income, BMI, and HbA1c. Also, age, income, BMI, and the number of complications cause vital differences in emotional well-being. | The study reveals that diabetic foot ulcers (DFUs) significantly affect the psychosocial well-being of patients, leading to diminished health-related quality of life (HRQOL). Patients reported feelings of isolation, anxiety, and depression, which often stemmed from their physical limitations and the perception of being a burden to their families. Social functioning was notably impacted, as many patients experienced reduced participation in community and family activities due to their condition. The study also highlighted that individuals with DFUs engaged in maladaptive behaviors, such as non-compliance with foot care routines, which were influenced by a lack of awareness about the risks associated with diabetes management. Furthermore, lower socioeconomic status and educational background were correlated with poorer psychosocial outcomes, reinforcing the need for targeted educational and support interventions to improve both physical care and emotional resilience among patients. Overall, the findings emphasize the complex interplay between physical health, emotional well-being, and social dynamics in the management of diabetic foot complications.  The study found that females with DFU had poorer HRQOL than males, particularly in physical functioning and roles related to physical health.  Contributing factors included forefoot lesions, larger ulcer size, advanced Wagner grade, and a higher frequency of unhealed ulcers among females, leading to worse mental and physical health outcomes. | This paper focuses on HRQOL in individuals with DFU and identified various demographic and clinical factors that influence HRQOL. It does not specifically explore the influence of psychosocial factors on the progression or recurrence of ulceration.  However, the paper indicates that psychosocial factors do influence the progression and recurrence of ulceration in diabetic patients. It highlights that emotional distress, such as anxiety and depression, can lead to poor adherence to foot care practices, increasing the risk of complications and ulcer recurrence. Additionally, the study suggests that factors such as social support and educational background significantly affect patients' ability to manage their condition effectively. The findings emphasize the importance of addressing psychosocial elements in diabetes care to prevent the worsening of foot ulcers and their associated complications. |
| 4. | (Ali Alzahrani & Sehlo 2013)  Saudi Arabia | Cross sectional Study  (Questionnaire) | 180  N with DFU= 60 | Health-Related Quality of Life (HRQL) Assessment (SF-36); Intrinsic/Extrinsic Religious Connectedness Scale; Clinical parameters of the patients; Physical and psychiatric examinations. | To explore the association between religious connectedness and health-related quality of life (HRQL) in people with and without diabetes and foot ulcers | Patients with diabetic foot ulcers had significantly lower health-related quality of life (HRQL) than those without ulcers and healthy controls, especially in physical functioning. Additionally, religious connectedness was positively correlated with improved HRQL, indicating that spiritual engagement may enhance well-being. | Individuals with DFU experience significant emotional and psychological consequences. This study indicates that patients with diabetic foot ulcers (DFU) face considerable emotional and psychological challenges, including impaired mental health, as evidenced by lower mental health scores compared to those without ulcers and healthy individuals. These patients are more likely to experience heightened levels of depression and anxiety, which negatively affect their overall quality of life. Additionally, many suffer from social isolation, further intensifying their emotional distress. However, the study also identifies religious connectedness as a beneficial coping mechanism, showing a positive correlation with improved mental health and health-related quality of life (HRQL). | The findings indicate that health-related quality of life (HRQL) is poor in diabetic patients with foot ulcers and worsens with increased severity, duration, and number of ulcers. The data also indicated a positive correlation between religious connectedness and HRQL, particularly in mental functioning. | The paper does not specifically explore whether psychosocial factors influence the progression or recurrence of diabetic foot ulcers (DFU). Instead, it focuses on the association between religious connectedness and health-related quality of life (HRQL) in patients with DFU, highlighting emotional and psychological consequences but not directly addressing how these factors may affect ulcer progression or recurrence.  However, the paper indicates that psychosocial factors do influence the progression and recurrence of ulceration in patients with diabetic foot ulcers. It highlights that emotional distress, anxiety, and depression can negatively affect patients' adherence to treatment and self-care practices, leading to poorer outcomes and increased likelihood of ulcer recurrence. Additionally, the study suggests that factors such as religious connectedness can play a positive role in improving mental health and overall quality of life, potentially helping to mitigate the adverse effects of psychosocial stressors. |
| 5. | (Antonin et al. 2021)  Italy | Randomized controlled study | 40  N with DFU= 20 | Measure of perceived Stress" (MPS) test | To examine if there is a correlation between infectious markers and emotional and functional stress factors in male and female populations | This study shows the existence of a close correlation between infectious markers and emotional and functional stress factors in both male and female populations. | This study highlights the existence of a close correlation between infectious markers and emotional and functional stress factors in both male and females. It specifically identifies a link between chronic conditions, such as chronic diabetic foot lesions (CDSFL), and behavioral changes measured through functional stress tests. The findings indicate that individuals with CDSFL often experience loss of control, irritability, confusion, depression, and anxiety. | N/A | N/A |
| 6. | (Beattie et al. 2014)  United Kingdom (Southwest of England) | Qualitative Study  (Interviews) | 15  N with DFU=15 | A semi-structured interview style was used Thematic analysis and the software ‘ATLAS.ti’ were used to aid data organization and coding | To examine the emotional and behavioural consequences of living with a heightened risk of re‐ulceration. | The study found that individuals living ulcer-free but at high risk for DFUs (past history) experience significant emotional distress. | Individuals experienced significant emotional distress, characterized by a lack of perceived control, negative emotions like fear and anxiety about re-ulceration, and feelings of guilt regarding past foot care neglect. Most participants reported a lack of perceived control in preventing further DFUs, which impacted their emotional and behavioral experiences. Many lacked sufficient knowledge about DFUs, leading to negative emotions such as fear of recurrence, uncertainty about future mobility, and concerns about limb loss. These feelings were compounded by issues of blame and guilt as Participants often felt guilty for not taking better care of their feet in the past, attributing their health issues to personal neglect.. | The primary psychosocial impact highlighted was a lack of perceived control over the risk of developing further diabetic foot ulcers (DFUs), with many participants feeling powerless even when ulcer-free. Factors influencing their behaviors included the lengthy healing process, treatment regimens, and frequent clinic visits, which restricted their daily lives and required time off work. Participants expressed concerns about sustaining positive behaviors after healing, often struggling to pace their activities. Women in particular noted that wearing orthotic shoes affected their femininity and challenged their identity. There was a notable gap between participants’ reported foot care practices and what they believed they should be doing. Many took calculated risks to maintain a semblance of normalcy, leading some to rush back into activities, which occasionally resulted in new ulcers.  Participants also found few opportunities to discuss their emotions with family or friends due to fears of stigma and not being understood. | Yes, the paper explored how psychosocial factors, particularly the lack of perceived control, influence the emotional and behavioral responses of individuals living with the threat of re-ulceration. It suggests that these emotional responses—such as fear, anxiety, and guilt—can impact self-care behaviors and may increase the risk of further diabetic foot ulcers (DFUs). The findings indicate that these psychosocial elements are critical to understanding the progression or recurrence of ulceration in this patient group. |
| 7. | (Bonner et al. 2017)  Texas, USA | Qualitative interview study | 12 | The conceptual model used in the current study was the self-regulatory model of illness representations. Phenomenological Methodology was used in this study. | To investigate how personal illness representations of type 2 diabetes affected the level of foot care knowledge and self-care strategies among African Americans adults. | Participants had limited knowledge of basic foot care and lower extremity disease, which negatively impacted their self-care practices. | The study found that most participants lacked a basic understanding of diabetic foot care and how uncontrolled type 2 diabetes can lead to lower extremity complications. it implies that a lack of understanding about foot care and potential complications can lead to feelings of anxiety and fear regarding health outcomes. Participants expressed concerns about their foot health, the risk of complications, and their ability to manage their condition effectively, which could contribute to emotional distress. The study suggests that improved education about T2DM and foot care could help alleviate some of these psychological burdens. | Participants were very cautious about maintaining foot hygiene, particularly keeping their feet clean. Many attributed poor blood flow to lifestyle factors, such as lack of exercise, poor diet, and inadequate hygiene. Throughout the interviews, participants expressed challenges in understanding their healthcare providers and felt they often did not receive enough information about their health status. Despite this lack of understanding, they generally placed a high level of trust in their providers. | This paper did not explore whether psychosocial factors influence the progression or recurrence of ulceration. Instead, it focused on the knowledge and self-care strategies related to type 2 diabetes and lower extremity disease among African Americans. While it discussed the importance of diabetes self-management education and the participants' understanding of foot care, it did not directly address how psychosocial factors might impact the recurrence of diabetic foot ulcers. |
| 8. | (Brod et al. 2015).  California, USA | Qualitative study (interviews and focus groups) | 70  N with DFU=70 | The transcripts were analyzed thematically; descriptive coding was used to identify emerging concepts. Data analysis was conducted using ATLAS.ti, a qualitative analysis software program. | To assess the burden and impact of Diabetic peripheral neuropathy pain (DPNP) symptoms on patient’s functioning and well-being. | Diabetic peripheral neuropathic pain (DPNP) significantly impacts patients' physical function, daily life, social interactions, and mental well-being. | This study underscores the significant burden of diabetic peripheral neuropathic pain (DPNP) on physical and psychological health-related quality of life. Participants reported that DPNP led to anxiety, fear, and stress, which contributed to irritability and, in some cases, depression. Approximately half of the respondents indicated difficulties in enjoying life, concentrating, and engaging socially, highlighting the pervasive impact of DPNP on overall well-being. Many expressed anxiety about their condition, including fears of potential amputations and the unpredictability of their symptoms. Feelings of depression were commonly linked to pain and limitations, further diminishing quality of life. Constant pain resulted in irritability that strained relationships with family and friends, while physical limitations and fear of injury led to social withdrawal, fostering loneliness and a lack of enjoyment. Additionally, pain disrupted sleep patterns, causing fatigue and negatively affecting mental well-being. Overall, the study emphasizes that DPNP significantly impacts emotional and psychological functioning, increasing the overall burden of the disease. | Participants reported significant limitations in walking, exercise, and recreational activities, along with reduced energy levels and productivity, which negatively affected their work and social relationships. | This paper did not specifically explore whether psychosocial factors influence the progression or recurrence of ulceration. Instead, it focused on the burden of diabetic peripheral neuropathic pain (DPNP) and its impact on patients' functioning and well-being. While it discussed various emotional and psychological consequences of living with DPNP, it did not directly address how these factors relate to ulceration outcomes. |
| 9. | (Chin et al. 2012)  Tawain | Cross-sectional Qualitative study (Structured Face to face Interviews with a standardised questionnaire) | 277  N with DFU= | Family support was measured using the Family APGAR (Neabel et al. 2000). Data on foot-exam practice, perceived self-efficacy and action cues were collected through the use of structured questionnaires. A Diabetic Foot Ulcer Health Belief Scale (DFUHBS), comprised of three subscales, was designed by the investigator. The data were analysed using SPSS software. | To identify the effects of health belief model factors on daily foot-exam practice among diabetes mellitus patients with peripheral neuropathy. | The study found that daily foot exam practices in patients with diabetic foot ulcers were significantly influenced by psychosocial factors, including self-efficacy and cues from family and healthcare providers. Key factors from the health belief model (HBM) linked to these practices included self-efficacy, action cues, perceived benefits, barriers, and threats, with foot-care recommendations, self-efficacy, and barriers as primary predictors. | The findings indicate that poor foot exam practices contribute to a sense of helplessness and reduced self-efficacy. | The study reveals that the psychosocial impact of diabetic foot ulcers (DFUs) significantly affects patients' behaviors and self-care practices, particularly regarding daily foot examinations. Many participants reported low engagement in foot-care behaviors, with a substantial portion never performing daily exams. The findings highlight that perceived barriers, such as physical limitations and lack of awareness about peripheral neuropathy, contribute to this under-practice. Conversely, positive action cues, such as advice from family, friends, or healthcare professionals, were found to significantly motivate participants to engage in foot care. Perceived self-efficacy also emerged as a crucial factor, with those who felt more confident in their ability to perform foot exams being more likely to do so. Overall, the study underscores the importance of addressing modifiable psychosocial factors to enhance self-management behaviors and improve health outcomes for patients with DFUs.  Participants in the study perceived high benefits and low barriers to foot care, along with a medium threat of foot ulcers. While only half received advice from family or healthcare professionals, most recognized the importance of foot self-care, likely due to early education from parents or teachers about hygiene and safe practices. Thus, self-care became part of their daily routine rather than being seen as disease-specific.The study also confirmed the link between self-efficacy and daily foot-exam practices, aligning with previous research. Additionally, it highlighted that perceived barriers are significant predictors of health behaviors, contributing new insights into how these barriers impact foot-care practices in diabetic patients. | The paper does not explicitly state that psychosocial factors influence the progression or recurrence of ulceration. Instead, it focuses on the impact of psychosocial factors on daily foot exam practices and self-care behaviors among patients with diabetic foot ulcers. However, it suggests that improved self-efficacy and positive action cues could potentially lead to better foot care, which may help prevent further complications, including ulcer progression or recurrence. |
| 10. | (Chin et al. 2019)  Tawain | Cross-sectional design (Structured face to interviews with a survey) | 199  N with DFU=199 | The study collected information on demographics, medical treatments, and diabetic foot ulcer (DFU) education. It assessed diabetes foot self-care behaviors prior to hospitalization using the valid and reliable Diabetes Foot Self-Care Behavior Scale (DFSBS). For diabetes-related foot ulcer self-management behavior, the Diabetes-related Foot Ulcer Self-Management Behavior Scale (DFUSMBS) was developed in two stages: initial item creation based on literature and clinical experience, followed by expert and patient feedback to finalize the scale. Knowledge of DFU deterioration warning signs was evaluated with the 12-item Warning Signs of Diabetic Foot Ulcer Deterioration Knowledge Questionnaire (WS-DFUD-KQ). | To determine prehospitalised diabetes-related foot ulcer (DFU) self-management behaviours and explore the factors associated with these behaviours. | Daily foot exam practices were suboptimal among patients with type 2 diabetes and peripheral neuropathy. | This paper primarily focuses on the impact of action cues, self-efficacy, and perceived barriers on daily foot exam practices in patients with type 2 diabetes and peripheral neuropathy. However, it is acknowledged that foot ulceration can significantly lower the quality of life in both physical and psychosocial domains, suggesting that living with DFUs may lead to increased anxiety, depression, and social isolation among affected individuals. | This study highlighted significant gaps in diabetes self-management among participants, particularly in monitoring blood glucose levels related to diabetic foot ulcers (DFUs). More than half showed little concern for their glycemic status, and fewer than 5% sought treatment within 24 hours of noticing a DFU. Many participants ignored pain and infection signs. Inadequate wound management practices, such as off-loading and keeping the wound dry, were observed, with less than one-fifth consistently reducing foot activity. Factors influencing DFU self-management included barrier beliefs, foot self-care behaviors, inadequate diabetes treatment, financial challenges, employment status, and patient education quality. Key barriers included a lack of awareness regarding timely self-management and the inconvenience of seeking care, emphasizing the need for enhanced patient education. | The paper did not specifically explore whether psychosocial factors influence the progression or recurrence of ulceration. While it acknowledges the importance of foot care in preventing diabetic foot ulcers, it does not address the direct relationship between psychosocial factors and ulceration outcomes. |
| 11. | (Coelho et al. 2009)  Brazil | Qualitative study (group and individual meetings) | 10  N with DFU=not reported | Thematic content analysis | To understand the social representations of the *diabetic foot* for people with type 2 diabetes mellitus. | The study identified two main social representations of diabetic foot among individuals with type 2 diabetes: perceptions of foot disease, marked by awareness of changes and threats, and foot care, which included future care concerns and feelings of guilt associated with non-care. These representations influence patients' behaviors and understanding of their condition, highlighting the importance of education and support in managing diabetes-related foot complications. | Patients with DFUs experience significant emotional and psychological challenges, including fear and anxiety over the risks of amputation and deteriorating health, leading to feelings of vulnerability. Many individuals also grapple with guilt for not following foot care recommendations, influenced by societal expectations and their sense of personal responsibility. The presence of foot complications negatively impacts self-esteem and self-image, as changes to their feet are perceived as a loss of independence. condition and adhere to preventive care practices.  The results show that the subjects seek hopes of not developing foot disease or controlling the situation, influenced by the representations of alterations and threats. When non-care occurs, the feeling of guilt emerges, since the subjects knew about the necessary care but did not use it. | Participants’ concerns about foot complications underscored the importance of care, as they acknowledged that while DM is chronic and incurable, they can take proactive steps to manage it.Preventive care was emphasized, with injuries seen as early warning signs of potential escalation. Family support played a crucial role, particularly when participants faced challenges in maintaining care due to physical limitations like mobility and vision.  The social representations contributed in the search for comprehension of how the subjects with diabetes mellitus build the knowledge that express their identity and guide their behavior, especially regarding the diabetic foot. | This paper did not specifically explore the influence of psychosocial factors on the progression or recurrence of ulceration in diabetic foot conditions. Instead, it focused on understanding the social representations of diabetic foot among individuals with type 2 diabetes mellitus, highlighting themes such as fear, guilt, self-esteem, and social isolation. While these emotional and psychological aspects are discussed, the paper does not directly address their impact on the progression or recurrence of foot ulcers. |
| 12. | (Cornell & Meyr, 2018)  Philadelphia | Cross-sectional Study  (A guided, physician-administered survey) | 15  N with DFU=15 | The survey consisted of both open-ended questions and a 10-point modified Likert scale for specific questions on a variety of potential patient concerns (Figure). The survey topics and potential patient concerns were derived from the authors’ collective clinical experiences. | 1. To explore the perceived concerns of patients with chronic wounds at risk for lower extremity amputation. 2. To provide wound care professionals with information that will benefit their approach to the education and treatment of patients at risk for amputation as well as lead to future investigations into the emotional and psychological state of patients with chronic lower extremity wounds. | Findings revealed anxiety and fear related to loss of mobility and self-sufficiency, with varied responses highlighting individual patient experiences. The highest concerns involved recurrence and functional impact, indicating the need for personalized patient education and support. | The authors observed that many patients experienced significant anxiety and fear. However, it was primarily related to concerns about loss of function and self-sufficiency following an amputation. | The highest levels of concern (mean measurements ≥ 7/10) had to do with recurrence, function, walking, and self-sufficiency. The lowest levels of concern (mean measurement ≤ 5/10) had to do with pain, shoe gear considerations, cost, and the cosmetic appearance of an amputation. | N/A. The objective of this investigation is to qualitatively and quantitatively explore the perceived concerns of patients with chronic wounds at risk for lower extremity amputation. |
| 13. | (Costa et al. 2020)  Canada | Qualitative studies (semistructured interviews, field notes, and the researcher’s journal were included in date collection’s procedures) | 30  N with DFU=30 | Constructivist grounded theory methodology | To uncover the factors influencing individuals’ ability to engage in self management of diabetic foot ulcer (DFU) and presenting a theoretical model depicting these. | The study found that aging, multiple chronic conditions, internal motivations, self-efficacy, and access to support significantly influence individuals' engagement in self-management of diabetic foot ulcers (DFUs). | Participants living with diabetic foot ulcers (DFUs) reported significant emotional and psychological consequences, including fear and anxiety about potential amputations and the effects on their daily lives. Many experienced guilt and shame for perceived failures in managing their condition, leading to a loss of independence as physical limitations restricted their normal activities. This often resulted in social isolation, exacerbating feelings of loneliness and depression. However, some participants maintained motivation and hope, driven by the desire to avoid further complications and regain their usual routines. | Participants faced numerous challenges in managing diabetes and diabetic foot ulcers (DFU) within their social lives, with socioeconomic factors significantly impacting their self-management abilities. They expressed a heightened awareness of the limitations imposed by aging and multiple chronic conditions, which affected their capacity to perform self-management tasks and control their lives. A strong desire to return to their normal routines motivated them to engage in self-management practices. Many participants defined a "normal" life as regaining the freedom to move and act without restrictions, which fueled their determination to preserve their feet and regain control. Self-management support, both informal (from family and friends) and formal (from healthcare providers), emerged as a crucial factor in achieving positive DFU outcomes. Participants often relied on family, particularly spouses and children, for assistance when self-efficacy or physical limitations hindered their self-management efforts. They valued the guidance and support received from healthcare providers, noting that having access to resources and a supportive environment was instrumental in maintaining their foot care and hope for healing. | The paper did not specifically explore the influence of psychosocial factors on the progression or recurrence of ulceration in diabetic foot ulcers (DFUs). Instead, it focused on identifying internal and external factors influencing individuals' engagement in self-management of DFUs. While emotional and psychological aspects, such as fear and motivation, were discussed, the primary emphasis was on understanding how these factors affect self-management practices rather than directly examining their impact on ulcer progression or recurrence. |
| 14. | (de Almeida et al. 2014).  Brazil | A qualitative study using semi-structured interviews grounded in a phenomenological framework. | 200  diabetic foot ulcers (N = 100) | A questionnaire assessing sociodemographic and clinical characteristics, and the Powerlessness Assessment Tool (PAT) for adult patients.  -SPSS and Microsoft Excel were used for Data Analysis | To assess feelings of powerlessness in patients with either venous or diabetic foot ulcers. | Patients with venous and [diabetic foot ulcers](https://www.sciencedirect.com/topics/nursing-and-health-professions/cyclorphan) had very strong feelings of powerlessness, but these feelings were significantly stronger in those with foot ulcerations. | Patients with diabetic foot ulcers reported significantly stronger feelings of powerlessness compared to those with venous leg ulcers, with 87% of diabetic foot ulcer patients expressing very strong feelings of powerlessness. The total PAT score was notably higher for patients with diabetic foot ulcers than for those with venous leg ulcers. | Venous and diabetic foot ulcers significantly diminish quality of life, impacting mobility, emotional well-being, and functional capacity. Many participants had low education levels, with over half being illiterate. This lack of education may have hindered understanding and adherence to self-care practices and treatment regimens, such as daily foot assessments, medication use, and proper dressing techniques. | The paper did not specifically explore the influence of psychosocial factors on the progression or recurrence of ulceration in DFUs. |
| 15. | (Habibu et al. 2022)  Nigeria | Cross-sectional study  (Structured Questionnaire) | 394  N with DFU= 57 | The **SF36** questionnaire was used to measure QOL. Data collected were analyzed using **SPSS**. | The aim of this study was to determine the QoL of persons with diabetic foot ulcers attending two major hospitals in northwestern Nigeria. | Individuals with diabetic foot ulcers (DFUs) experienced significantly poorer health-related quality of life (QoL) compared to those without DFUs, affecting all domains measured by the SF-36 questionnaire. | Patients with DFUs reported a significant decline in their overall quality of life, affecting their physical, mental, and emotional well-being. Individuals with DFUs often experienced difficulties in social interactions and activities, contributing to feelings of loneliness and emotional distress. The burden of managing DFUs, including treatment costs and potential loss of income, added to the emotional strain and stress experienced by patients.The challenges of living with DFUs, including chronic pain and mobility issues, led to emotional instability and increased family tension.  Summary: The attached paper outlines the emotional and psychological consequences of living with diabetic foot ulcers (DFUs), noting a significant decline in patients' quality of life across physical, mental, and emotional dimensions. DFUs increase the risk of complications, leading to heightened anxiety and stress regarding health outcomes. Patients often face social isolation, which exacerbates feelings of loneliness and emotional distress. Additionally, the financial burden of managing DFUs, including treatment costs and potential income loss, contributes to emotional strain. Chronic pain and mobility issues further result in emotional instability and family tension, collectively impacting the mental health of individuals with DFUs. | Using the SF36 instrument to assess health-related quality of life (HRQL), it was revealed that DFU patients scored worse across all domains compared to non-ulcer patients. The most notable differences were in physical functioning, role limitations due to physical health, and emotional health, indicating that patients with foot ulcers experienced significantly poorer HRQL in all eight domains and median summary scores. | While it did discuss the impact of DFUs on patients' physical, mental, and emotional well-being, this paper did not specifically investigate how these emotional or psychosocial factors might affect the progression or recurrence of ulcers. The emphasis was on the overall burden of DFUs on QoL and associated health outcomes. |
| 16. | (Arisandi et al. 2018)  Indonesia | Pilot Randomized Controlled Trial | 60  N with DFU=60 | **Self-efficacy** was measured using an adapted self-efficacy instrument.  **Psychological distress** was assessed with a modified version of the Psychological Distress in Diabetes Scale (DDS).  **Self-care** was evaluated using the Summary of Diabetes Self-Care Activities (SDSCA).  **Quality of life** was measured with the Wound Quality of Life Short Form (QOL). The secondary outcome focused on wound closure or changes in DFU status, assessed using the Diabetic Foot Ulcer Assessment Scale (DFUAS). | This study aimed to evaluate psychological aspects by conducting an intervention consisting of understanding wellbeing. | Patients with DFU that received treatment on the understanding of wellbeing appeared to exhibit similar changes in psychological factors and acceleration of wound healing, compared to those who did not receive this intervention, except in the distress parameter at the second follow up. | This paper highlights several emotional and psychological consequences of living with diabetic foot ulcers (DFUs), including significant psychological distress and challenges with self-efficacy that undermine patients' confidence in managing their health. It notes a decline in overall quality of life, particularly in emotional well-being, as well as social isolation due to difficulties in interactions, leading to feelings of loneliness. Additionally, patients experienced increased anxiety about their health outcomes, especially regarding the risk of ulcer recurrence and associated complications. | The study indicated that individuals with DFUs struggled with self-efficacy, impacting their confidence in managing their health and treatment. The overall quality of life for patients with DFUs was negatively affected, with emotional well-being being particularly compromised. | While it acknowledged the impact of DFUs on patients' lives, this paper did not delve into the direct relationship between psychosocial factors and the recurrence or progression of ulcers. |
| 17. | (Jelsness-Jørgensen et al. 2011).  Norway | Cross-sectional study  (Questionnaire) | 257  N with DFU =127 | The Short Form-36 (SF-36) was used to asses HRQOL.  The ankle-brachial index (ABI) was used to determine peripheral vascular disease. The Wagner classification of diabetes foot ulcers was used to assess the level and extent of the ulcers. | The primary aim of this study was to describe HRQOL in DO and to identify sociodemographic and/or clinical variables that significantly influence HRQOL. Secondary aims were to investigate the effects of foot ulcers on HRQOL, by comparing patients with and without diabetic foot ulcers (DFU – DO). | Health-related quality of life differs signiﬁcantly between disease subgroups when measured with Short Form-36. | The study reveals significant emotional and mental health impacts associated with diabetic foot ulcers (DFU) in diabetes patients. Those with DFUs reported markedly lower mental health scores on the Short Form-36 (SF-36), indicating a decline in emotional well-being compared to those without foot complications. Gender differences were evident, with women in both groups experiencing poorer mental health outcomes. While comorbidities like cardiovascular disease were common, they did not significantly affect mental health in the DFU group; however, they were linked to lower scores in the diabetes outpatient group. Additionally, emotional issues hindered patients' daily activities, contributing to reduced vitality and social functioning. The study underscores the importance of psychosocial support to mitigate feelings of isolation and enhance mental health among diabetes patients. | This study confirms significant differences in health-related quality of life (HRQOL) among disease subgroups using the Short Form-36 (SF-36). Patients with diabetic foot ulcers (DFU) experienced a major negative impact on 7 out of 8 subscales compared to the diabetes outpatient group. The study links HRQOL scores to sociodemographic and clinical data, highlighting that patients with DFUs have much poorer HRQOL than those without foot ulcer issues. DFU patients showed a more pronounced reduction in HRQOL compared to those with diabetes alone, indicating that long-term diabetes complications are key predictors of both physical and mental HRQOL. P atients who have developed diabetic foot ulcers reports much poorer health-related quality of life than compared to diabetes outpatients. This study identifies subgroups of outpatients even before developing late complications, by linking their HRQOL scores to sociodemographic and clinical data. Patients who have developed foot ulcers because of diabetes impairment have much poorer HRQOL when compared with patients without foot ulcer problems; however, cardiovascular comorbidity and neuropathy are both major predictors of lower HRQOL scores even in earlier stages of disease. | This paper did not delve into the direct relationship between psychosocial factors and the recurrence or progression of ulcers. However, it reports that patients with DFUs had a more pronounced reduction in HRQOL than patients with DM only, indicating that long-term DM complication is a major predictor of both physical and mental HRQOL and immediate change in HRQOL scores. |
| 18. | (Khunkaew et al. 2019)  Thailand | Cross-sectional study (Questionnaire) | 502  N WITH DFU= 41 | HRQOL was evaluated using the Thai version of Diabetes-39 | The aim of this study was to determine the demographic and clinical predictors of health-related quality of life among people with type 2 diabetes mellitus (T2DM) in Northern Thailand. | Individuals with type 2 DM in Northern Thailand reported low health-related quality of life, with diabetic foot ulcers and smoking identified as significant predictors of lower HRQOL. | This study reveals significant emotional and mental health impacts. Participants reported low overall HRQOL scores, indicating a considerable emotional burden, particularly in the domains related to anxiety and worry. The presence of DFUs emerged as a key predictor of poor HRQOL across multiple dimensions, highlighting how physical complications can lead to increased anxiety and social isolation, thereby negatively affecting mental health. Furthermore, lower income levels were associated with greater anxiety and worry, underlining the influence of socioeconomic factors on mental health outcomes. | The study on health-related quality of life (HRQOL) among individuals with type 2 diabetes mellitus (T2DM) in Northern Thailand highlights significant psychosocial impacts and associated behaviors. Participants reported low HRQOL scores, particularly in areas related to anxiety, worry, and social burden, indicating that living with T2DM leads to considerable emotional distress and social isolation. The presence of diabetic foot ulcers (DFUs) exacerbated these issues, as individuals faced not only physical discomfort but also heightened anxiety and limitations in daily activities. | While it acknowledged the impact of DFUs on quality of life, this paper did not delve into the relationship between psychosocial factors and ulcer recurrence or progression. |
| 19. | (Kim & Han, 2020)  Korea | Cross-sectional study  (Survey) | 131  N WITH DFU= 131 | Demographic, disease-related and laboratory characteristics were collected. | To examine the level of self-care behaviours among patients with diabetic foot ulcers and identify factors related to their self-care behaviours. | Perceived family support was the main factor affecting diabetes man-agement, and diabetic foot care was greatly affected by the experience of diabetic education. | The study highlights significant emotional and mental health impacts associated with DFU. Patients with DFUs often experience feelings of distress, discomfort, and regret regarding their wounds, which can lead to emotional turmoil and a diminished quality of life. The chronic nature of DFUs, coupled with the risk of complications such as amputation, contributes to heightened anxiety and stress levels among patients.  In addition, the DFU patients in this study felt a moderate level of diabetes-related stress and used an emotion-focused coping style rather than a problem-focused coping one. | The study on patients with diabetic foot ulcers (DFUs) reveals significant psychosocial impacts and associated behaviors that influence self-care. Patients often face emotional challenges, including stress and anxiety, stemming from the chronic nature of their condition and the potential for severe complications such as amputation. The experience of diabetic education is shown to positively correlate with improved self-care behaviors, highlighting its critical role in empowering patients. Additionally, perceived family support emerges as a vital factor, enhancing patients' ability to manage their diabetes effectively and engage in proactive foot care. Conversely, higher levels of perceived stress are linked to poorer self-care practices, indicating that emotional well-being directly affects health outcomes. The findings emphasize the need for comprehensive interventions that address both the psychological aspects of living with DFUs and the practicalities of diabetes management, suggesting that family involvement and education can significantly improve self-care behaviors and overall quality of life for these patients. | While it acknowledged the impact of DFUs on patients' QOL this paper did not delve into the direct relationship between psychosocial factors and the recurrence or progression of ulcers. |
| 20. | Kazan Kizilkurt et al. 2020)  Turkey | Cross-sectional Study (interviews and surveys) | 65  N WITH DFU= 65 | Short Form 36, The Trinity Amputation and Prosthesis Experience Scale, Coping Attitudes Evaluation Scale, Multidimensional Scale of Perceived Social Support, Rosenberg Self-Esteem Scale, and Amputee Body Image Scale were administered as questionnaires. Stepwise linear regression analysis was conducted to assess the factors predicting quality of life. | The purpose of this study was to identify clinical and psychosocial factors that predict an individual’s subjective quality of life after having undergone a lower limb amputation secondary to diabetic foot ulcer | After lower extremity amputation due to diabetic foot ulcers, quality of life is significantly impacted by factors such as satisfaction with the prosthesis, body image, self-esteem, and coping strategies. | The study highlights that DFU patients often experience significant psychological distress following amputation, including feelings of anxiety, depression, and a distorted body image. These emotional challenges can lead to decreased self-esteem and social isolation, further complicating the adjustment process.  . The study revealed that quality of life (QOL) is significantly influenced by factors such as depression, anxiety, body perception, self-esteem, perceived social support, and coping strategies. QOL negatively correlates with depression, anxiety, body image issues, activity limitations, and dysfunctional coping, while positively correlating with social support, prosthetic satisfaction, self-esteem, and problem-focused coping. Regression analysis identified prosthetic satisfaction, body perception, problem-focused coping, dysfunctional coping, and self-esteem as key predictors of the physical component of QOL. For the mental component, body perception and coping strategies were the main predictors. Overall, poor body image, low self-esteem, dysfunctional coping, and dissatisfaction with prosthetics strongly indicate reduced quality of life. | ThIs study found that both physical and mental quality of life (QOL) were significantly lower in individuals after lower limb amputation compared to the general population. Key factors influencing QOL included stump and phantom pain, comorbid medical conditions, and the level of prosthetics used.  . The experience of phantom and stump pain further complicates their adjustment, negatively affecting both physical and mental quality of life. Coping strategies are crucial; individuals who utilize problem-focused coping tend to adapt more successfully, while those relying on dysfunctional coping mechanisms experience poorer psychosocial outcomes. Additionally, perceived social support plays a vital role in facilitating emotional resilience, as those who feel supported are better equipped to manage their condition. The findings underscore the importance of addressing these psychosocial factors in rehabilitation programs, suggesting that tailored interventions focusing on emotional well-being and coping strategies can significantly improve the overall quality of life for amputees. | While it discussed the emotional and psychosocial challenges faced by individuals post-amputation, the paper did not address the direct impact of these factors on the recurrence of DFUs. |
| 21. | (Kuang et al. 2021)  China | Prospective cross-sectional study  (Questionnaire) | 98  N WITH DFU= 98 | Social support: The multidimensional scale of perceived social support (MSPSS) questionnaire.  Classification of foot ulcer: Wagner ulcer classification  Psychological resilience: Connor-Davidson resilience scale (CD-RISC)  Self-efficacy: The diabetes management self-efficacy scale (DMSES).  Quality of life: 36-item short-form health survey (SF-36). | To examine the role of psychological resilience in QoL and self-efficacy in DFU patients | Psychological resilience significantly impacts the self-efficacy and quality of life of patients with DFUs. | The study highlights the importance of psychological resilience, showing that those with higher resilience report better self-efficacy and quality of life. Additionally, perceived social support plays a crucial role, as patients who receive more support tend to exhibit greater resilience, positively influencing their emotional well-being. Furthermore, older age and low resilience are identified as risk factors contributing to poorer emotional and psychological outcomes in DFU patients.  This study examines the emotional and mental health impacts of diabetic foot ulcers (DFUs) on patients, emphasizing the role of psychological resilience. Patients with DFUs often experience significant psychological distress, including low self-efficacy, anxiety, and depression, which adversely affect their quality of life (QoL). The findings indicate that individuals with low psychological resilience tend to have poorer mental health outcomes, characterized by diminished self-esteem and increased feelings of helplessness. Those with high psychological resilience report better emotional well-being, enhanced coping abilities, and greater satisfaction with their treatment and prosthetics. Furthermore, the study highlights the importance of social support in fostering psychological resilience, suggesting that patients who receive adequate support tend to have better mental health outcomes. Overall, the emotional and mental health challenges faced by DFU patients underscore the necessity for targeted interventions that promote resilience and support to improve their overall well-being and QoL. | The study identified several risk factors impacting self-efficacy and quality of life (QoL) in participants, including low psychological resilience, older age, lower perceived social support, and higher HbA1c levels. Participants with high resilience were more likely to be employed and reported significantly more social support.  The research emphasizes the crucial role of social support in enhancing psychological resilience, indicating that patients who receive greater support are more likely to experience positive mental health outcomes. Overall, the findings suggest that fostering resilience and providing adequate social support can significantly mitigate the psychosocial burdens associated with DFUs, ultimately improving patients' emotional health and quality of life. | While it addressed various emotional and psychological aspects related to the condition, this paper did not directly examine their effects on ulcer progression or recurrence. |
| 22. | (Livingstoneet al. 2011).  Australia | Qualitative Study  (interviews) | 5  N WITH DFU=5 | Theoretical coding and grounded theory | The aim of this study was to allow amputees to describe their experiences of amputation and to generate grounded theory that will lead health professionals towards a more comprehensive understanding of the realities of post-amputation life. | This study identified three core categories—"imposed powerlessness," "adaptive functionality," and "endurance"—that encapsulate the experiences of individuals with diabetes-related amputations. | The study found that medical errors and a lack of awareness regarding the risks of diabetic amputations led to feelings of uncertainty and fear. Participants experienced grief, loss, and shock after surgery, but they gradually developed hope through a coping process that demonstrated their resilience and ability to regain control over their lives despite the challenges of a chronic disease. .Participants in the study reported a profound sense of powerlessness due to ongoing complications and negative interactions with the healthcare system, which led to uncertainty and fear. Following their amputations, they experienced significant grief and loss, struggling with changes in mobility and daily life. Initially, many felt shock, anger, and disbelief, accompanied by emotional turmoil. However, they adapted their movements and social roles to cope with their new realities, showcasing resilience amidst ongoing challenges. | Social support played a crucial role in fostering resilience, as most participants had supportive partners, while one individual lived in a supportive environment with a caregiver. | This paper did not specifically explore whether emotional or psychosocial factors influence the progression or recurrence of diabetic foot ulcers (DFUs). Instead, it focused on the experiences of individuals who underwent diabetes-related amputations, examining their emotional and psychological responses to amputation, such as feelings of powerlessness, grief, and the process of adaptation. |
| 23. | Mendonça et al. (2022)  Portugal | Cross-sectional Study  (Questionnaires) | 62  N WITH DFU=20 | •Integrated Palliative care Outcome Scale (IPOS) •EuroQol-5D three-level version (EQ-5D-3L) | The aim of this study was to characterize palliative care needs in people under diabetic foot surveillance using the Integrated Palliative care Outcome Scale (IPOS) and EuroQol-5D three-level version (EQ-5D-3L) and to assess differences between those with and without a DFU. | Individuals with active DFUs reported significantly higher levels of anxiety and emotional distress, alongside physical symptoms such as pain and weakness. | The study found that individuals with active DFU exhibited a higher level of emotional and psychological distress, with 95% of DFU patients feeling anxious or worried compared to 55% of those without DFU (p = 0.002). Communication challenges were evident, as only 10% of patients with active DFU felt they could share their feelings with family and friends as often as desired, compared to 45% of those without DFU (p = 0.006). Overall, the findings indicate that patients with active DFU face significant emotional and psychological challenges, coupled with a reduced ability to communicate their feelings.  Patients with active diabetic foot ulcers (DFUs) experienced significantly higher levels of anxiety and emotional distress compared to those without ulcers, with 95% reporting feelings of anxiety. Many also struggled with depression and found it difficult to share their feelings with others, as only 10% felt they could express their emotions freely. | The presence of DFUs adversely impacted their quality of life, leading to feelings of isolation and reduced social interaction due to mobility challenges. Additionally, physical symptoms like pain and fatigue exacerbated their emotional distress, further affecting their overall well-being. | While it acknowledged the impact of DFUs on quality of life and emotional well-being, this paper did not investigate the direct relationship between these factors and ulcer progression or recurrence. |
| 24. | (Nemcová et al. 2017)  Visegrad countries | Cross-sectional Study  (Questionnaires) | 525  N WITH DFU=525 | Quality of life: The standardised generic questionnaire World Health Organization Quality of Life-BREF was used.  Classification of foot ulcer: Wagner ulcer classification | To identify the quality of life of patients with diabetic foot ulcers in the Visegrad countries. | Socio-demographic factors and clinical characteristics significantly impact the quality of life in patients with diabetic foot ulcers across Visegrad countries. Notably, older age, longer duration of diabetes and ulcer treatment are associated with a decreased quality of life. | The findings of this study indicate significant differences in QoL across all domains—physical, psychological, social, and environmental—among participants from different Visegrad countries. Older age was linked to poorer QoL, particularly in psychological and social areas.  The study on the quality of life in patients with diabetic foot ulcers (DFU) across the Visegrad countries reveals significant emotional and mental health impacts associated with the condition. Patients frequently experience feelings of helplessness and hopelessness, largely due to the chronic nature of their illness, which can lead to depression, especially when compounded by fears of amputation.  Furthermore, the emotional burden is reflected in the correlation between the severity of foot pain and quality of life across various domains. Those suffering from pain reported poorer scores in physical, psychological, and social aspects of life.  Many patients reported feelings of social isolation and dependence on others for daily activities, which adversely affected their self-esteem and overall mental health. The emotional strain is compounded by chronic pain and the fear of potential amputations, leading to increased anxiety and depressive symptoms  Furthermore, the study found that patients who received adequate information about their condition and treatment experienced improved psychosocial well-being, suggesting that education can empower patients and foster more proactive engagement in their care.  Overall, the findings indicate that the emotional and mental health impacts of diabetic foot ulcers are profound, necessitating a holistic approach in their management that addresses not just the physical but also the psychological and social dimensions of care.  . | Negative correlations were identified between QoL and factors such as age, duration of diabetes, and length of ulcer treatment. Clinical characteristics, including Wagner classification, frequency of ulcers, presence of peripheral vascular disease, and pain, also significantly impacted QoL.. Those living alone faced worse QoL across all domains due to limited social interactions, while those with partners reported better QoL. Additionally, patients informed about their treatment had significantly higher QoL scores across all dimensions compared to those without information.  The research highlights that many patients reported a lower quality of life linked to physical limitations imposed by DFU, which restricts mobility and independence, thereby exacerbating feelings of social isolation.  The study also emphasizes the role of social support, indicating that patients living alone experienced worse mental health outcomes compared to those with a partner or family support, underlining the importance of social connections in mitigating the psychological impacts of DFU.  Behaviors such as reduced participation in social activities and reliance on caregivers were common, highlighting the detrimental effects of DFU on social interactions and support systems. | The paper does not explicitly state that psychosocial factors influence the progression or recurrence of ulceration. However, The paper indicates that psychosocial factors significantly influence the progression and recurrence of diabetic foot ulcers (DFU). It highlights that patients experiencing higher levels of anxiety, depression, and social isolation are more likely to have poorer health outcomes, including the progression of their ulcers. The emotional burden associated with living with DFU can lead to reduced adherence to treatment and self-care practices, further contributing to the risk of recurrence. Additionally, the study suggests that social support and adequate patient education can mitigate these psychosocial challenges, potentially leading to better management of the condition and a lower likelihood of ulceration reoccurrence. |
| 25 | (Kragh Nielsen et al. 2022)  Denmark | Qualitative Study  (Interviews) | 5  N WITH DFU=5 | Interpretative Phenomenological Analysis (IPA). | This study aims to explore the thoughts of patients with diabetic foot ulcers regarding leg amputation. | The study reveals four key themes in patients' thoughts on leg amputation due to diabetic foot ulcers: solitude, social perceptions, self-efficacy, and the balance of limitations and opportunities. Even without an imminent amputation, patients struggle with these complex feelings, highlighting the need for healthcare professionals to address the topic sensitively to encourage open communication. | This study explores the emotional and mental health impacts of potential leg amputation in patients with diabetic foot ulcers. It reveals that the prospect of amputation generates a range of complex emotions, including fear, anxiety, and feelings of isolation. Participants often perceive amputation as a taboo subject, leading to reluctance in discussing their thoughts and concerns with healthcare professionals or loved ones. This silence exacerbates their emotional burden, as they navigate feelings of guilt and self-blame, particularly regarding their perceived responsibility for their health. | The study highlights significant psychosocial impacts and associated behaviors among patients with diabetic foot ulcers facing potential leg amputation. Participants experience emotional isolation due to the stigma surrounding amputation, which is often viewed as a taboo topic, making it difficult for them to share their thoughts and fears with others. This isolation exacerbates feelings of anxiety and depression, as patients grapple with concerns about their self-worth and societal perceptions post-amputation. Many express a desire for support and connection, indicating that discussions with peers or support groups could alleviate some of their emotional burdens. Additionally, participants reflect on their expected self-efficacy, with some feeling empowered to cope with the challenges of amputation, while others fear dependency and loss of independence. Overall, the findings suggest that the psychosocial effects of potential leg amputation are profound, underscoring the importance of supportive communication and resources to help patients navigate their experiences.  Overall, the findings highlight the stigma surrounding leg amputation and the barriers it creates for open communication.  Additionally, the participants express worries about how amputation would affect their social interactions and personal identity, fearing negative judgments from strangers. Despite these challenges, some participants also identify potential opportunities for increased independence through the use of prosthesis and express a desire for better communication and support from healthcare providers. Overall, the findings underscore the need for healthcare professionals to foster open dialogues about amputation, addressing both the psychological and social implications to better support patients’ mental health and emotional well-being. | The paper does not explicitly state that psychosocial factors influence the progression or recurrence of ulceration. However, it discusses how emotional and mental health impacts, such as anxiety, depression, and feelings of isolation, can affect patients' self-care behaviors and adherence to treatment. These factors may indirectly contribute to the progression or recurrence of diabetic foot ulcers, suggesting a potential link between psychosocial well-being and physical health outcomes. Overall, while the study emphasizes the importance of addressing psychosocial elements, it does not provide direct evidence on their influence on ulceration progression or recurrence. |
| 26. | (Nyanzi et al. 2014)  Uganda | Cross-sectional design  (Questionnaire) | 219  N WITH DFU=118 | Quality of life standardized instrument for Indian diabetes patients (QOLID) developed by Nagpal et al. (2012). | To determine if factors associated in the literature on quality of life in diabetes apply to diabetic patients in Uganda. | The study assessed quality of life across five domains and found significant variations in role limitation due to physical health, influenced by age, education, diabetic foot ulcer status, retinopathy, and alcohol consumption (P < 0.05). While sex and type of diabetic treatment showed some correlation, their significance was lower (P < 0.5). | This study reveals that the quality of life for diabetic patients is significantly influenced by their emotional well-being, with factors such as age, education level, and the presence of diabetic complications, like foot ulcers, playing crucial roles. Patients often experience stress and anxiety related to managing their condition, which can lead to feelings of helplessness and frustration. The emotional burden is compounded by the need for lifestyle changes and adherence to treatment protocols, which can be overwhelming for many. Additionally, the study notes that lower educational attainment is associated with poorer quality of life, as less educated patients may struggle to understand and manage their diabetes effectively. This lack of understanding can exacerbate feelings of inadequacy and diminish their overall mental health. Ultimately, the findings underscore the importance of addressing both the physical and emotional aspects of diabetes management to improve the overall quality of life for patients. | The presence of complications, such as diabetic foot ulcers, further exacerbates these emotional burdens, leading to decreased physical endurance and limitations in daily activities. Patients with lower levels of education experience greater difficulties in understanding and adhering to treatment regimens, which can contribute to feelings of inadequacy and reduced self-esteem. The study also notes that social factors, including support from family and friends, play a vital role in coping with diabetes; patients who lack a strong support network may struggle more with their mental health. | The paper does not explicitly state that psychosocial factors influence the progression or recurrence of ulceration. However, it discusses the emotional and mental health challenges faced by diabetic patients, particularly those with complications like foot ulcers. The findings suggest that emotional distress and inadequate understanding of diabetes management can adversely affect patients' adherence to treatment and self-care practices, which may indirectly impact the progression or recurrence of diabetic ulcers. While the study acknowledges the importance of psychosocial factors in the overall quality of life for diabetic patients, it does not establish a direct link between these factors and ulceration outcomes. |
| 27. | (Pedras et al. 2018)  Portugal | Cross-sectional study  (Questionnaire) | 202  N WITH DFU=202 | •Socio demographic and clinical questionnaire •Hospital Anxiety and Depression Scale  •Barthel Index  •Short-Form Health Survey 36 | The goal of this study was to analyze the relationships between anxiety, depression symptoms, and functionality as predictors of quality of life, in patients with diabetic foot ulcer taking in consideration clinical variables. | Findings indicate that anxiety, depression, and functionality levels significantly predict both mental and physical quality of life (HRQoL) in patients with DFU. The results underscore the importance of psychological interventions and early rehabilitation programs to enhance functionality and overall HRQoL, advocating for a multidisciplinary treatment approach that addresses both physical and psychological factors. | This study highlights significant emotional and mental health impacts associated with diabetic foot ulcers (DFU). Patients indicated for amputation surgery exhibited high levels of anxiety and depression, with nearly 60% showing clinical symptoms of anxiety and 38% experiencing depressive symptoms. The psychological burden of facing a potential amputation contributes to heightened anxiety, as patients grapple with fears about their health outcomes and future mobility. Additionally, the study found that these emotional challenges are closely linked to functionality; those with higher levels of functional dependence reported worse mental health outcomes. The findings suggest that the mental health implications of living with DFU are profound, with psychological morbidity negatively affecting health-related quality of life (HRQoL). Recognizing and addressing these emotional and mental health impacts is crucial for improving overall patient care and outcomes. The authors advocate for integrating psychological interventions into the clinical management of DFU to alleviate symptoms of anxiety and depression, thereby enhancing patients' quality of life. | This study reveals significant psychosocial impacts associated with diabetic foot ulcers (DFU), particularly in patients scheduled for amputation surgery. Many participants experienced high levels of anxiety and depression, which were found to correlate with their functional limitations and dependency in daily activities. The emotional distress stemming from their condition often led to social withdrawal and reduced engagement in meaningful activities, compounding feelings of isolation and helplessness. Furthermore, the research indicates that patients with higher anxiety and depression symptoms were less likely to adhere to self-care practices, negatively influencing wound healing and increasing the risk of recurrence. | Yes, the study suggests that psychosocial factors significantly influence the progression and recurrence of diabetic foot ulcers (DFU). It highlights that high levels of anxiety and depression can lead to poorer adherence to self-care behaviors, which in turn increases the risk of wound healing complications and recurrence. The study emphasizes that psychological morbidity adversely affects health-related quality of life (HRQoL), further suggesting that emotional distress can hinder effective management of the condition. By addressing these psychosocial factors, the research advocates for improved patient outcomes and reduced risks of ulcer progression and recurrence. |
| 28. | Pereira et al. (2014)  Brazil | Cross-sectional Study (Exploratory, descriptive, analytical design)  Questionnaire | 50  N WITH DFU=50 | •Socio demographic and clinical questionnaire  • Powerlessness Assessment Tool for adult patients (PAT). | The purpose of this study was to assess feelings of powerlessness in patients with diabetic foot ulcers. | Patients with DFUs experience significant powerlessness and emotional distress, which correlate with reduced treatment adherence and poor wound healing. The authors recommend including powerlessness assessments in intervention planning to lessen the impact of diabetic foot ulcers on daily life. | Many participants reported high levels of anxiety and low self-esteem, which were closely linked to their chronic condition and the physical challenges it presents  The findings also reveal that a majority of participants reported moderate to high levels of powerlessness, indicating a perceived lack of control over their health outcomes. This emotional distress is compounded by physical symptoms, such as wound odor and exudate, which can further exacerbate feelings of shame and isolation. The study emphasizes the necessity for healthcare professionals to address both the physical and psychological needs of patients, advocating for interventions that not only focus on wound care but also provide emotional support to help mitigate the profound mental health challenges associated with diabetic foot ulcers. | The presence of symptoms such as wound odor and exudate not only affects physical health but also contributes to social withdrawal and a diminished self-image, further isolating patients. This emotional turmoil can lead to maladaptive behaviors, including decreased adherence to treatment and self-care practices, as individuals grapple with feelings of hopelessness. | The paper does not explicitly state that psychosocial factors influence the progression or recurrence of ulceration. However, the study indicates that psychosocial factors, particularly feelings of powerlessness and emotional distress, can influence the progression and recurrence of diabetic foot ulcers. It highlights that patients experiencing significant emotional challenges, such as anxiety and low self-esteem, may be less likely to adhere to treatment and self-care practices. This non-adherence can lead to poor wound healing and an increased risk of ulcer recurrence. |
| 29. | (Perrin et al. 2022)  Netherlands | Observational Cross-sectional study  (Questionnaire) | 304  N WITH DFU= 299 | •Dutch translation of the RAND® 36-Item Short Form Health Survey (SF-36) | The aim of this study was to assess HRQoL and determine factors associated with HRQoL in people with diabetes at high risk of foot ulceration. | The study found significantly lower HRQoL in patients compared to the general population and those with existing foot ulcers. Key factors associated with reduced HRQoL included the use of walking aids, non-Caucasian ethnicity, unemployment, higher BMI, and younger age. Psychosocial factors like emotional distress also impacted HRQoL, emphasizing the need for comprehensive care to address both physical and emotional well-being. | This study highlights the significant emotional and mental health impact of diabetic foot ulcers on individuals at high risk for ulceration. It found that participants exhibited reduced health-related quality of life (HRQoL), particularly in the physical domains, with lower scores in areas such as General Health, Role Physical, and Physical Function. Although mental health scores were relatively higher compared to physical scores, the participants still faced challenges related to anxiety and depression, often exacerbated by their physical limitations and the fear of potential ulcer recurrence. | The study reveals that individuals at high risk of diabetic foot ulcers experience considerable psychosocial impacts, significantly affecting their health-related quality of life (HRQoL). Participants reported lower scores in physical health domains, which correlated with increased feelings of disability and limitations in daily activities. Factors such as younger age, non-Caucasian ethnicity, and unemployment further exacerbated these psychosocial issues, indicating a need for targeted interventions. The findings suggest that individuals’ behaviors, such as reduced physical activity and social engagement, stem from both physical limitations and the emotional toll of living with diabetes-related complications. | The paper does not explicitly state that psychosocial factors influence the progression or recurrence of ulceration. However, Yes, the paper suggests that psychosocial factors do influence the progression and recurrence of diabetic foot ulcers. It highlights that emotional distress, anxiety, and feelings of disability can lead to decreased adherence to self-care practices and treatment regimens, which are critical for preventing ulcer recurrence. The study emphasizes that individuals experiencing higher levels of psychosocial stress may be more susceptible to complications, indicating a significant link between psychosocial well-being and the management of diabetic foot health. |
| 30. | (Polikandrioet al. 2020)  Athens | Cross-sectional Study  (Questionnaire) | 180  N WITH DFU= 180 | •“Self‐rating Depression/Anxiety Scale‐ Zung” (SDS/SAS) •Multidimensional Scale of Perceived Social Support (MSPSS) | The purpose of this study was to explore the impact of anxiety and perceived social support on depression of DFU patients well as patients' characteristics associated with depression. | Patients with diabetic foot ulcers (DFUs) experienced low quality of life, especially in physical and emotional aspects, with factors like age, education, and self-care adherence significantly influencing their well-being. This underscores the need for integrated care addressing both physical and psychosocial aspects of DFU management. | The findings indicate that patients with diabetic foot ulcers (DFUs) are more susceptible to depression and anxiety compared to those without foot complications. Notably, higher perceived social support was linked to lower levels of depression, suggesting that enhanced social support could improve health outcomes for DFU patients. This relationship may be bidirectional, where increased support reduces depression and vice versa. Key factors associated with higher depression levels included older age, being single or divorced, having only primary education, being a pensioner, having comorbid conditions, and current smoking.  This study finds that individuals with DFUs often experience high levels of anxiety and depression, with 13.8% and 20.0% of participants reporting high levels of anxiety and depression, respectively. These psychological factors are closely linked to the overall quality of life (QoL) of patients, as those with higher anxiety and depression scores reported significantly lower QoL across various domains, including physical functioning and emotional well-being. The emotional burden is compounded by the physical limitations imposed by DFUs, which restrict mobility and daily activities, leading to feelings of helplessness and social isolation. | The study reveals a profound psychosocial impact of diabetic foot ulcers (DFUs) on patients, significantly affecting their quality of life (QoL). Many participants reported low levels of physical functioning and emotional well-being, which are critical to daily life and social interactions. | Yes, the paper by Polikandrioti et al. indicates that psychosocial factors significantly influence both the progression and recurrence of diabetic foot ulcers (DFUs). It highlights that higher levels of anxiety and depression are associated with poorer adherence to self-care practices, which are crucial for preventing ulcer progression and recurrence. |
| 31. | (Ratliff & Rovnyak 2021)  USA | Prospective, descriptive study  (Questionnaire) | 111  N WITH DFU= 24 | The Wound-QoL questionnaire | To describe health-related quality of life (HRQoL) using the Wound-Quality of Life (Wound-QoL) questionnaire for those individuals referred to an academic medical center wound clinic. | Individuals with DFUs experience significant emotional distress, severely impacting their HRQoL, particularly in psychological and daily life domains. The Wound-QoL questionnaire indicated that emotional concerns, such as worry and frustration about healing, have the greatest impact on HRQoL, suggesting its usefulness in evaluating wound effects on patients' well-being. | This study reveals that individuals with DFUs often face emotional challenges such as anxiety, worry, and frustration regarding their condition and its treatment.  The emotional subscale of the Wound-QoL showed the highest mean scores, indicating that psychological factors play a crucial role in health-related quality of life (HRQoL) for these patients. Participants reported heightened anxiety about the potential worsening of their condition and the burden of treatment, which can lead to social withdrawal and feelings of helplessness. This emotional turmoil not only affects the individuals psychologically but also impacts their daily activities and overall well-being, underscoring the need for comprehensive care that addresses both the physical and emotional dimensions of managing diabetic foot ulcers. | The findings suggest that patients with DFUs frequently experience a decline in their health-related quality of life (HRQoL), particularly in emotional and everyday life domains. This emotional distress can lead to maladaptive behaviors, including reduced adherence to self-care practices and increased social withdrawal, as patients may feel embarrassed or dependent on others due to their wounds. The study underscores the importance of addressing these psychosocial factors in clinical settings to improve patient outcomes, emphasizing that emotional support and interventions aimed at enhancing self-efficacy are crucial for better managing the physical and psychological burdens associated with DFUs. | Yes, the paper indicates that psychosocial factors significantly influence the progression and recurrence of diabetic foot ulcers (DFUs). It highlights that emotional distress, particularly anxiety and depression, can adversely affect patients' adherence to self-care practices, which are essential for preventing ulcer progression and recurrence. |
| 32. | (Reina-Bueno et al. 2021)  Spain | Observational cross-sectional study  (Questionnaire) | 50  N WITH DFU= 50 | Spanish version of the generic SF-36 questionnaire but with one additional dimension of study: health change. | To investigate whether there is a different impact on quality of life according to gender and to establish the physiological parameters that these patients have in relation to diabetes-related complications. | DFUs significantly affect patients' emotional and mental well-being, with women reporting lower health-related quality of life (HRQoL) than men. Complications like neuropathy and peripheral artery disease worsen psychological distress, emphasizing the need for integrated care that addresses both physical and psychosocial factors to enhance patient outcomes. | The study highlights the emotional and mental health impact of diabetic foot ulcers (DFUs) on patients, revealing that individuals with DFUs often experience significant psychological distress, including anxiety and depression. It was found that women, in particular, reported lower health-related quality of life (HRQoL) scores in emotional and mental health dimensions compared to men. This disparity may be attributed to sociocultural factors, as women often bear additional burdens from household responsibilities alongside managing their health. The presence of complications such as peripheral neuropathy and peripheral artery disease further exacerbates feelings of helplessness and frustration, affecting patients' overall well-being. Consequently, the study emphasizes the need for targeted psychological support and interventions to improve the emotional resilience of individuals dealing with DFUs, particularly among women who may face unique challenges in coping with their condition.  While the physical health parameters of diabetic patients were similar across genders, adult women consistently exhibited lower quality of life levels than men. | The emotional toll of managing DFUs is compounded by the physical limitations imposed by the condition, fostering feelings of frustration and helplessness. Additionally, sociocultural factors, such as gender roles and responsibilities, further influence how patients cope with their health challenges. Overall, the findings suggest that understanding the psychosocial dimensions of living with DFUs is crucial for developing effective management strategies that enhance both emotional well-being and adherence to medical advice. | Yes, the paper indicates that psychosocial factors significantly influence the progression and recurrence of diabetic foot ulcers (DFUs). It highlights that emotional distress, particularly anxiety and depression, can negatively impact patients' adherence to self-care practices, which are essential for preventing complications and recurrence. |
| 33. | (Ribu et al. 2007)  Norway | Cross-sectional study  (Questionnaire) | 6251  N WITH DFU= 127 | The SF-36 Health Survey | To describe health-related quality-of-life (HRQL) in patients with diabetic foot ulcers by comparing their HRQL with that of a sample from the general population without diabetes (general population) and a subgroup with diabetes (diabetes population), and to examine the differences between groups by sociodemographic characteristics and lifestyle factors | Individuals with DFUs reported significantly lower health-related quality of life (HRQoL) than both the diabetes population and the general population, especially in physical health. Emotional and mental health challenges, worsened by social isolation and anxiety, were common among DFU patients. This highlights the need for integrated care addressing both physical and psychosocial aspects, as well as the importance of considering sociodemographic factors in diabetes research. | This study reports that patients with diabetic foot ulcers exhibited poorer mental health as indicated by the mental health summary scale (MCS) compared to the diabetes population (p < 0.05) and these patients also reported significantly lower social functioning than the diabetes population.  Patients with DFUs face heightened feelings of physical and emotional limitations, which contribute to a pervasive sense of distress and a diminished capacity to engage in daily activities. The research indicates that these patients often report increased levels of pain and discomfort, exacerbating their mental health challenges. The prolonged healing process associated with DFUs can lead to anxiety and uncertainty about recovery, further affecting their emotional well-being.  The emotional burden of living with DFUs often leads to increased anxiety and depression, particularly among older men who are more likely to live alone and face social isolation. | The study reveals that patients with diabetic foot ulcers (DFUs) experience significant psychosocial challenges that adversely affect their quality of life. The emotional burden of living with DFUs often leads to increased anxiety and depression, particularly among older men who are more likely to live alone and face social isolation. These psychosocial factors contribute to maladaptive behaviors, such as reduced social interaction and lower engagement in self-care practices, which can hinder treatment adherence and recovery. The findings suggest that the physical limitations imposed by DFUs not only restrict daily activities but also strain personal relationships and foster feelings of being a burden to others | Yes, the paper indicates that psychosocial factors significantly influence the progression and recurrence of diabetic foot ulcers (DFUs). It highlights how emotional distress, such as anxiety and depression, can negatively impact adherence to self-care practices, which are crucial for preventing complications and ulcer recurrence. The study underscores the need to consider these psychosocial aspects in treatment and management strategies to improve patient outcomes and reduce the likelihood of reoccurrence. |
| 34. | (Salomé et al. 2013)  Brazil | Cross-sectional (clinical, analytical, descriptive study) | 80  N WITH DFU=40 | The Powerlessness Assessment Tool for Adult Patients (PAT) and the Herth Hope Index (HHI) | To assess feelings of powerlessness and hope for cure in patients with chronic venous leg ulcers (VLUs) and diabetic foot ulcers (DFUs). | The results suggest that patients with DFUs had stronger feelings of powerlessness regarding their condition and less hope of recovery compared with patients with VLUs. High scores on the Powerlessness Assessment Tool indicate a profound psychosocial impact, suggesting that these emotional challenges may hinder treatment adherence and overall quality of life for affected individuals. | Many patients experience low self-esteem due to the chronic nature of their condition, which often leads to pain, mobility limitations, and social isolation. The presence of symptoms like wound odor and exudate further exacerbates these feelings, contributing to a sense of loss of control over their lives. The majority of participants reported high scores on the Powerlessness Assessment Tool, indicating strong feelings of helplessness. This emotional burden not only affects their quality of life but also complicates their treatment adherence and overall health outcomes.  The mean Powerlessness Assessment Tool (PAT) score was significantly higher for DFU patients (53.3±9.6) compared to VLU patients (34.3±7.7; p=0.001), indicating greater feelings of powerlessness. Conversely, the Hopefulness Index (HHI) was lower for DFU patients (16.5±16.5) than for VLU patients (27.5±27.5; p=0.001), reflecting lower levels of hope for healing. Overall, DFU patients exhibited stronger feelings of powerlessness and less hope regarding their wounds than those with VLUs. | The presence of DFUs and VLUs has a negative  impact on patients and their family members; they  are associated with pain, fear of leg amputation and  decreased functional status, which affects activities  of daily living (such as dressing and walking), intensifying the dependency of these patients.  Emotional distress is linked to various psychosocial behaviors, such as reduced engagement in daily activities and social interactions due to pain, mobility limitations, and the stigma associated with visible wounds. Patients frequently report feelings of anxiety and depression, which can hinder their motivation to adhere to treatment regimens and self-care practices. The presence of unpleasant symptoms like wound odor and exudate further contributes to these negative emotions, leading to increased frustration and social withdrawal. | The paper does not directly establish a causal relationship between psychosocial factors and the progression or recurrence of venous leg ulcers (VLUs). However, it suggests that the emotional and psychological distress associated with feelings of powerlessness can negatively impact patients' engagement in self-care and treatment adherence. This lack of adherence may, in turn, contribute to poorer healing outcomes and the potential for ulcer recurrence. The study emphasizes the importance of addressing psychosocial factors to improve overall patient management, which could indirectly influence the progression or recurrence of VLUs. |
| 35. | (Searle et al. 2007)  UK | Qualitative study (Semi-strucutured interviews) | 25  N WITH DFU= 13 | N/A | To explore the experience of foot ulceration and treatment from the perspective of patients and the podiatrists who treat them. | The study found that patients with diabetic foot ulcers experience significant emotional distress, with high levels of anxiety and depression impacting their quality of life. | The results of this study indicate that foot ulcers and their treatment significantly affect patients' mobility, independence, and social lives, often leading to feelings of anger, fear, depression, helplessness, boredom, and diminished self-esteem. Depression was most prevalent following the diagnosis of a foot ulcer and during periods of persistent or recurring problems. Many patients expressed distress over their ulcers, with several experiencing episodes of depression. Common causes included slow healing, recurrent ulceration, medication use, lifestyle restrictions, and loss of independence. The threat of amputation also contributed significantly to anxiety and depression. Additionally, patients reported that their ulcers affected their self-esteem and social lives, leading to feelings of boredom and frustration due to the necessity of resting their foot.  The document explores the emotional and mental health impact of diabetic foot ulcers, emphasizing the profound psychological distress experienced by patients. Many individuals report feelings of anxiety, depression, and isolation due to their condition, which can significantly alter their quality of life. The ongoing nature of ulceration often leads to a sense of helplessness, as patients struggle with the unpredictability of healing and the limitations imposed by their foot problems. This emotional burden is compounded by concerns about mobility, independence, and the potential for social stigma. The qualitative findings highlight the need for healthcare providers to acknowledge these emotional challenges and to offer supportive care that addresses both the physical and psychological aspects of living with diabetic foot ulcers. Such an integrated approach is crucial for improving patient outcomes and fostering a better overall sense of well-being. | The study highlights the significant psychosocial impact of diabetic foot ulcers on patients, revealing that individuals frequently experience heightened levels of anxiety, depression, and feelings of isolation. These emotional challenges often lead to maladaptive behaviors, such as withdrawal from social interactions and decreased engagement in self-care practices, which can hinder recovery and exacerbate the condition. Patients commonly report a loss of control over their lives, contributing to a pervasive sense of helplessness and frustration. The emotional strain can also affect their relationships, as they may feel like a burden to family and friends. | Yes, the paper indicates that psychosocial factors significantly influence the progression and recurrence of diabetic foot ulcers. It highlights that emotional distress, such as anxiety and depression, can negatively affect adherence to self-care practices, which are essential for preventing complications and ulcer recurrence. The authors suggest that understanding and addressing the psychosocial aspect of foot ulceration may lead to better adherence and improve clinical outcomes. |
| 36. | (Searle et al. 2008)  UK | Cross-sectional Study  (Questionnaire) | 68  N WITH DFU=22 | The assessment of beliefs was conducted with an adapted version of the Revised Illness Perception  Questionnaire (IPQ-R; Moss-Morris et al., 2002). | To compare the beliefs of 22 patients with diabetic foot ulcers and 22 age- and gender-matched patients with diabetic retinopathy, and 22 age- and gender-matched controls with type 2 diabetes but without either complication. | Patients with foot ulcers held a greater belief in personal control of diabetes, but perceived treatment control was lower than that of diabetic controls without serious complications. Patients with foot ulcers also demonstrated less illness coherence than patients with retinopathy and diabetic controls and also perceived their diabetes to be more cyclical in nature.Future interventions should consider how the complications associated with diabetes may affect patients’ beliefs and subsequent emotional and behavioral responses to the disease. | Patients with foot ulcers demonstrated a stronger belief in their personal control over diabetes, though their perceived treatment control was lower compared to diabetic patients without serious complications (p < .05). While foot ulcer patients reported greater emotional beliefs than those with retinopathy and control patients, these differences were not statistically significant. The scores for emotional beliefs were modest, failing to capture the psychological morbidity typically associated with diabetes or the psychosocial impact of living with a diabetic foot ulcer or retinopathy. | Patients frequently perceive their illness as cyclical and unpredictable, which contributes to a lack of coherence in understanding their condition and may hinder effective self-management behaviors. | Yes, the paper suggests that psychosocial factors significantly influence the progression and recurrence of diabetic foot ulcers. It highlights that emotional distress, such as anxiety and depression, can adversely affect patients' adherence to self-care practices, which are crucial for preventing complications and ulcer recurrence. The findings suggest that Differences were found in diabetic patients’ beliefs according to their complications. Future interventions should consider how the complications associated with diabetes may affect patients’ beliefs and subsequent emotional and behavioral responses to the disease. |
| 37. | (Selçuk Tosun et al. 2022)  Turkey | Qualitative study  (Interviews) | 14  N WITH DFU=14 | n/a | To evaluate the effects of foot ulcers on male patients diagnosed with type-2 diabetes, foot care and treatment, and the difficulties, experiences, feelings, and perspectives of male patients regarding foot ulcers. | Participants had knowledge of foot care and diabetes management, but their self-care practices were inadequate. Well-managed diabetes and external factors were found to be critical determinants of the development of foot ulcers, and DFUs were found to negatively affect the participants physically, mentally, and socially. | The emotional and mental health impact of diabetic foot ulcers (DFUs) is profound, as revealed through participant interviews. Many individuals expressed feelings of anger, sadness, and hopelessness, particularly when reflecting on their past and the progression of their condition. The initial neglect of symptoms—attributed to a lack of awareness and daily foot care—often led to a sense of regret when they finally recognized the severity of their ulcers. The physical limitations imposed by DFUs, including pain and difficulty walking, further exacerbated feelings of frustration and dependency, resulting in increased anxiety and stress. Participants also reported emotional neglect and fatigue, stemming from the challenges of managing their condition while trying to meet familial obligations. Financial difficulties and the perceived inability to control their health contributed to a sense of despair and loss of social functionality. Overall, the psychological burden associated with DFUs significantly affects individuals’ quality of life, underscoring the need for comprehensive support that addresses both physical and emotional health challenges.  .A subtheme titled "Psychological effects" emerged from 78.57% of participants, highlighting emotions such as rage, anger, fear of job loss, hopelessness, and anxiety, stemming from their loss of social functionality. Many felt they could not meet their own or their family's needs, leading to feelings of regret and despair about their past and future.  •Participants felt anger, sadness, and regret when they thought of the past and hopelessness, stress, and anxiety when they thought of the future. The participants also expressed that they felt regret at their negligence. | The study reveals significant psychosocial impacts associated with diabetic foot ulcers (DFUs), highlighting how their development and management affect patients' emotional and social well-being. Participants reported various initiating factors for DFUs, including ill-fitting shoes and minor injuries, often ignoring early warning signs until the condition worsened. This negligence reflects a lack of daily foot care routines, which is compounded by delays in seeking medical attention, often triggered only by unbearable pain or external pressure from family. The physical consequences of DFUs, such as pain and dependence on others, lead to feelings of frustration and anger, while social effects include financial difficulties and loss of employment, contributing to increased dependency and diminished quality of life. Despite receiving education on diabetes management, many struggled to implement self-care practices due to financial constraints and a lack of motivation, indicating that effective interventions must address both the practical and emotional barriers to self-care in this population.  Another subtheme, "Social effects," was identified from 64.2% of participants, encompassing issues like dependence on others, loss of pleasure, financial difficulties, and disability retirement. Participants reported experiencing financial hardships and having to close their businesses, increasing their reliance on family for daily activities. Physical challenges included pain, insomnia, and frequent hospitalizations due to foot ulcers and related complications. Some participants attributed their conditions to negligence or improper practices and noted that prolonged inactivity led to weight gain and reduced social engagement. | The study/paper does not explicitly state that psychosocial factors influence the progression or recurrence of diabetic foot ulcers (DFUs). However, it suggests that factors such as lack of awareness, neglect of self-care, and emotional responses like regret and hopelessness can contribute to the development and worsening of DFUs. Participants reported that they often delayed seeking care until their condition became severe, indicating that psychosocial barriers may hinder timely intervention. Furthermore, the discussion highlights how poor management of diabetes and the presence of multiple comorbidities can complicate recovery and increase the likelihood of ulcer recurrence. Overall, while psychosocial factors are acknowledged as influential in the context of self-care and treatment adherence, the text does not directly link them to the progression or recurrence of DFUs. |
| 38. | (Watson-Miller 2006)  Bermuda | Qualitative study  (Interviews) | 6  N WITH DFU=6 | A semi-structured interview topic guide was prepared based on social support questions adapted from Antoni et al. (2007) to suit participants with a DF. Phenomenological methodology was used for data analysis. | To explore participants’ experience with diabetic foot ulceration (DFU) in Bermuda. | DFUs were found to significantly impact patients' psychosocial well-being, leading to heightened anxiety, feelings of dependence, and changes in self-care behaviors. Despite strong family support, patients experienced embarrassment and a loss of control, underscoring the need for holistic care that addresses both physical and emotional aspects of their condition. | This study explores the emotional and mental health impact of diabetic foot ulcers (DFUs) on patients in Bermuda, revealing significant psychological distress associated with the condition. Participants reported pervasive anxiety and worry, primarily related to concerns about prolonged healing and the fear of amputation. This anxiety was compounded by feelings of dependence on others for daily activities, which altered family roles and dynamics, leading to feelings of being a burden. Despite these challenges, participants did not express feelings of social isolation, citing strong family support. However, the presence of unpleasant odors from the ulcers contributed to embarrassment and impacted their self-esteem. | Participants expressed worry about the healing process and the potential for amputation, which heightened their stress levels. This emotional turmoil often led to changes in behavior, including reduced engagement in self-care practices and a sense of losing control over their lives. Although participants reported increased reliance on family members for assistance, they also acknowledged the support received, which mitigated feelings of social isolation. | Yes, the paper indicates that psychosocial factors significantly influence the progression and recurrence of diabetic foot ulcers. The emotional distress experienced by patients, particularly anxiety and feelings of dependence, can adversely affect their self-care behaviors, which are crucial for healing and preventing recurrence. |
| 39. | (Palaya et al. 2018)  Australia | Qualitative study (interviews) | 8  N WITH DFU=8 | A semi-structured interview topic guide was as adapted from existing literature to explore the participants' perceptions of social support related to living with diabetic foot ulcers (DFU). A Hermeneutic Phenomenological Approach and Hybrid Thematic Analysis was used for data analysis. | To explore the perception of social support in individuals living with a diabetic foot in order to influence future service delivery in management of similar individuals. | DFUs were found to significantly affect patients' psychosocial well-being, causing anxiety, stress, and feelings of isolation. While some participants benefited from strong social support, many struggled with emotional challenges and practical barriers, underscoring the necessity for comprehensive care that addresses both mental health and social needs. | Participants reported feelings of isolation, anxiety, and stress associated with their condition, particularly regarding the fear of complications such as amputation. Many individuals expressed a desire for emotional self-efficacy, often internalizing their feelings rather than sharing them with support networks, leading to isolation and stress. This self-reliance sometimes resulted in depressive symptoms. Many participants were reluctant to engage with mental health services, viewing their emotional struggles as personal failings and dismissing the need for psychological support, which could worsen their emotional state and hinder recovery. | While some acknowledged the importance of support networks, they felt that family and friends often did not understand the severity of their condition, contributing to feelings of being a burden.  The study highlighted the importance of social support, noting that while some participants relied heavily on family and friends, others felt burdened by their need for assistance. This emotional turmoil was linked to decreased self-efficacy and reluctance to seek professional help, ultimately affecting their overall well-being.  This reluctance to seek help sometimes resulted in crises that could exacerbate their condition. Participants also faced practical challenges, such as transport needs for accessing care, which affected their social functioning. While some found support in their relationships, others felt burdened by their circumstances, leading to a cycle of dependency and diminished self-esteem.  Positive interactions with healthcare professionals offered reassurance and emotional support, highlighting the need for empathetic communication in medical settings. Additionally, financial concerns added to the emotional burden, as participants struggled to afford necessary medical supplies and treatments, limiting their social engagement and further isolating them from their communities. | The findings suggest that poor psychosocial well-being can lead to neglect in self-care practices and increase the risk of complications, including ulceration and recurrence. Therefore, addressing these psychosocial factors is essential for effective management and prevention of DFU. |
| 40. | (Putri et al. 2021)  Indonesia | Cross-sectional correlational design | 201  N WITH DFU=  201 | Diabetic Foot Ulcer Scale–Short Form (DFS-SF): This scale measures quality of life specific to patients with DFU. It consists of 29 items grouped into six domains: leisure, physical health, dependence/daily life, negative emotions, concern about the ulcers/feet, and concerns about ulcer care. Responses are rated on a 5-point Likert scale.  Body Investment Scale (Body Image Domain): This questionnaire assesses body image and includes 20 items divided into three domains, focusing specifically on the body image aspect for this study. Responses are also rated on a 5-point Likert scale, where higher scores indicate more positive feelings about the body. | The overall aim of the study is to examine the role of body image as a mediator in the relationship between gender and quality of life (QoL) among patients with diabetic foot ulcers (DFU) in Indonesia. | The study found significant gender differences in quality of life and body image among patients with DFUs, with women reporting poorer outcomes in both areas. Body image was identified as a full mediator between gender and quality of life, explaining 39.13% of the variance, indicating that body image concerns significantly affect women's overall quality of life in the context of DFU. | This paper reveals that individuals with DFUs often experience emotional distress, including shame, embarrassment, and reduced self-esteem, with women reporting a more negative body image than men. This negative perception is linked to physical limitations caused by DFUs, such as the inability to wear preferred footwear, affecting their sense of femininity and attractiveness. | Participants reported feelings of isolation and reluctance to engage in social and religious activities due to body image concerns, which heightened their emotional distress. The inability to participate in culturally significant practices exacerbated feelings of guilt and low self-worth among women, significantly impacting their overall QoL as they manage their condition alongside psychological challenges. | The paper primarily focuses on the relationship between body image, gender, and quality of life among patients with diabetic foot ulcers. While it discusses the emotional and psychosocial impact of living with DFU, it does not explicitly report on the influence of psychosocial factors on the progression or recurrence of ulceration. The emphasis is more on how these factors affect quality of life rather than their direct impact on ulceration outcomes. |
| 41. | Vileikyte et al. 2009, 2007 & 2004  UK & USA | Cross-sectional longitudional design | 338  N WITH DFU=  At baseline, 14.7% of the participants had an active foot ulcer. Additionally, during the 9-month follow-up, 14% of the study participants developed new foot ulcers. | This study utilized several instruments and measures to assess various aspects related to diabetic peripheral neuropathy (DPN) and depressive symptoms:  **Neuropathy Disability Score (NDS):** This clinical measure assesses the severity of neuropathy based on neurological function.  **Vibration Perception Threshold (VPT):** A measure of large fiber dysfunction used to evaluate neuropathy severity.  **Neuropathy and Foot Ulcer-specific Quality of Life Instrument (NeuroQoL):** This instrument includes scales to assess:  Neuropathic pain and/or paresthesiae  Symptoms of reduced feeling in the feet  Unsteadiness  Restrictions in activities of daily living (ADL)  Social self-perception  **Hospital Anxiety and Depression Scale (HADS):** Specifically, the HADS-D subscale was used to measure depressive symptoms, focusing on anhedonia and the absence of positive affect. | The aim of this study is to determine whether diabetic peripheral neuropathy (DPN) is a risk factor for depressive symptoms and to examine the potential mechanisms underlying this relationship. Specifically, the study seeks to explore the temporal relationships between DPN severity, associated symptoms (such as pain and unsteadiness), psychosocial consequences (like restrictions in activities of daily living and social self-perception), and the development of depressive symptoms over time in patients with DPN. | This study found that diabetic peripheral neuropathy (DPN) significantly predicts increased depressive symptoms over time, with unsteadiness being the strongest associated factor. Additionally, psychosocial consequences, such as restrictions in activities of daily living and changes in social self-perception, mediated the relationship between DPN symptoms and depressive outcomes, highlighting the complex interplay between physical and emotional health in affected individuals. | This paper highlights that DPN significantly contributes to depressive symptoms among patients, with various psychosocial factors mediating this relationship.  Key findings indicate that individuals with DPN often experience feelings of unsteadiness, pain, and limitations in their ability to perform activities of daily living (ADL), all of which are linked to increased depressive symptoms over time. The study demonstrates that unsteadiness is particularly impactful, as it not only contributes to physical challenges but also diminishes patients' self-worth and social roles, leading to feelings of inadequacy and isolation.  Psychosocial consequences arise from the restrictions imposed by DPN, influencing patients' social self-perception. Many individuals report feeling like a burden to family and friends, which exacerbates feelings of depression and anxiety. The inability to engage in social activities due to physical limitations further contributes to emotional distress.  The study emphasizes that these emotional challenges are not merely a byproduct of physical symptoms; rather, they are interwoven with the experience of living with DPN and DFU. | Social factors significantly impacted the psychological well-being of participants with diabetic peripheral neuropathy (DPN). Many felt isolated due to mobility limitations, which hindered social interactions and community participation, leading to decreased social support and increased feelings of loneliness and depression. Stigma surrounding diabetes and its complications further diminished self-esteem and social self-perception, creating barriers to seeking connections and support. This interplay highlights the need for fostering supportive social networks and promoting community engagement as vital elements of comprehensive care for those facing diabetes-related challenges. | The paper does not specifically report on the influence of psychosocial factors on the progression or recurrence of ulceration. Instead, it focuses on the relationship between diabetic peripheral neuropathy (DPN), depressive symptoms, and psychosocial consequences. While it highlights the impact of psychosocial factors on emotional well-being, it does not directly address how these factors may affect ulceration outcomes. |
| 42. | (Monami, et al. 2008)  Italy | Prospective cohort design | 80  N WITH DFU=80 | The study utilized the following instruments and measures:  Geriatric Depression Scale (GDS): To assess depressive symptoms among participants.  Greenfield Index of Disease Severity: To evaluate the overall severity of disease in patients.  Ulcer Area Measurement: To track the size of the ulcers over time.  Ankle-Brachial Index: To assess blood flow in the lower limbs.  Vibration Perception Threshold: To measure sensory function and neuropathy. | The aim of the study was to assess the role of depressive symptoms in the healing and recurrence of diabetic foot ulcers (DFUs) in elderly type 2 diabetic patients. It sought to explore the relationship between emotional well-being and ulcer outcomes, focusing on how depression impacts healing processes and the likelihood of ulcer recurrence. | The study found that depressive symptoms significantly hinder the healing of DFUs and increase the risk of recurrence, with patients scoring ≥10 on the Geriatric Depression Scale having a threefold higher risk of non-healing. Additionally, higher levels of depression were associated with poorer overall health and increased comorbidity, emphasizing the need for integrated psychological care in managing DFUs. | The paper highlights that individuals with DFUs often experience significant psychological distress, which can impair their quality of life and hinder the healing process.  Key emotional consequences include heightened levels of depression, particularly in elderly patients with chronic ulcers. Patients with depressive symptoms exhibited increased anxiety and feelings of hopelessness, exacerbating their overall health condition. | Psychosocially, the presence of DFUs can lead to social isolation, as individuals may withdraw from social interactions due to embarrassment or fear of judgment regarding their condition. The inability to engage in daily activities and maintain social roles further contributes to feelings of inadequacy and diminished self-worth. The paper underscores that these emotional and psychosocial challenges are not merely secondary effects of physical symptoms but represent a complex interplay that can directly affect healing outcomes.  The authors advocate for a holistic approach to treatment that includes psychological screening and intervention, recognizing the critical role that emotional well-being plays in the management of DFUs. | Yes, the paper reports that psychosocial factors, specifically depressive symptoms, significantly influence the recurrence of diabetic foot ulcers (DFUs). Higher scores on the Geriatric Depression Scale were associated with an increased risk of ulcer recurrence, indicating that emotional well-being can impact the progression of ulceration in diabetic patients. The study highlights the importance of addressing these psychosocial factors in the management of DFUs. |
| 43. | Goodridge (2003/2006) Canada | Cross-sectional, comparative design | 104  N WITH DFU=104 | The study utilized several instruments and measures to assess health-related quality of life (HRQOL) and related factors:  SF-12: A generic health status questionnaire measuring physical and mental health components.  Cardiff Wound Impact Scale (CWIS): A condition-specific instrument designed to evaluate the impact of diabetic foot ulcers on HRQOL.  Verbal Analogue Scales: Used to assess overall quality of life across various domains, including physical, emotional, spiritual, and intellectual well-being.  Demographic and Health Condition Surveys: Collected data on participants' background, health conditions, and treatment regimens. | The aim of the study was to examine health-related quality of life (HRQOL) in adults with diabetic foot ulcers (DFUs). Specifically, it sought to compare HRQOL between two groups: individuals with current DFUs and those with healed DFUs, as well as to assess how these groups' HRQOL compared to population norms for the general population and individuals with diabetes and hypertension. . | The study found that individuals with current DFUs experienced significantly lower health-related quality of life (HRQOL) scores, particularly in the physical domain, compared to those with healed DFUs and normative populations. Emotional well-being was also compromised, with participants reporting high levels of anxiety and frustration related to their condition, underscoring the substantial psychosocial impact of living with DFUs. | This paper highlights that emotional well-being is significantly affected in individuals with diabetic foot ulcers (DFUs), as participants reported feelings of frustration, anxiety, and social isolation due to mobility restrictions. Results from the Cardiff Wound Impact Scale (CWIS) indicate that patients often feel anxious about their wounds and frustrated with the healing process, leading to a diminished sense of well-being. The findings suggest that the psychological distress associated with DFUs can exacerbate feelings of helplessness and reduce adherence to essential self-care practices. | One-third of participants reported consistently facing difficulties with everyday tasks, while a similar percentage reported no issues. Challenges included mobilizing outside the home and finding appropriate footwear, with nearly half experiencing frequent difficulties in getting out and maintaining contact with family and friends. Although many did not express a desire to withdraw from social interactions or fear of worsening their wounds, well-being scores were notably low, indicating significant impairment in physical health-related quality of life (HRQOL). Individuals with current diabetic foot ulcers (DFUs) reported worse physical HRQOL than those with healed DFUs, highlighting the detrimental impact of active ulcers on physical well-being. | Yes, the paper reports that psychosocial factors, particularly depressive symptoms, influence the progression and recurrence of diabetic foot ulcers (DFUs). Higher levels of anxiety and frustration associated with living with DFUs are linked to poorer outcomes, indicating that emotional well-being plays a critical role in ulcer healing and recurrence. |
| 44. | (Costa, 2011)  Nebraska | Qualitative study ( interviews) | 10  N WITH DFU=10 | The study utilized qualitative semi-structured interviews as the primary instrument for data collection. Participants were asked open-ended questions to elicit detailed accounts of their experiences living with diabetic foot ulcers. Additionally, demographic questionnaires were employed to gather background information on the participants, such as age, education level, and medical history. The interviews were audio-recorded, transcribed, and analyzed using thematic analysis to identify common themes and perceptions. | The aim of the study was to investigate patients' perceptions of living with a diabetic foot ulcer and to explore how educational programs influence their experiences and understanding of diabetes and its complications. The study sought to understand the emotional, psychological, and social impacts of living with diabetic foot ulcers among individuals in Western Nebraska. | This study found that patients living with DFUs experience significant emotional distress, characterized by sadness, anger, and anxiety, which negatively impacts their social interactions and overall quality of life. Additionally, a lack of adequate diabetes education contributes to feelings of isolation and helplessness, underscoring the need for comprehensive support and educational programs. | This study highlights the profound emotional challenges faced by patients with diabetic foot ulcers (DFUs), including feelings of sadness, anger, anxiety, and depression due to the limitations of their condition. Participants frequently reported pervasive sadness linked to physical restrictions and the impact of diabetes on daily life, often feeling hopeless and losing interest in previously enjoyed activities. Depression was common, with many receiving antidepressant treatment. Anger also surfaced, directed at the disease, healthcare systems, and societal perceptions. Additionally, anxiety about potential complications, such as amputations, contributed to a chronic state of stress among participants. | The study reveals that living with diabetic foot ulcers (DFUs) often leads to social isolation, as participants experienced a decline in social interactions and recreational activities, resulting in loneliness. The emotional toll of DFUs also strained familial and social relationships, with many feeling like a burden to their families, which heightened their distress. Additionally, inadequate diabetes education and feelings of denial impeded effective coping strategies; while some sought self-education, many felt unprepared to manage their condition, perpetuating negative emotions and diminishing their quality of life.  All ten participants expressed feelings of hopelessness and a sense of emptiness. They reported that their relationships with family and friends deteriorated as they felt like burdens. Sadness was frequently linked to limitations in physical and social activities. | Yes, the paper discusses the influence of psychosocial factors on the progression and recurrence of diabetic foot ulcers. It highlights that emotional distress, such as sadness and anxiety, can negatively impact self-management and adherence to treatment, potentially leading to worsening conditions or reoccurrence of ulcers. The study emphasizes the need for addressing these psychosocial factors to improve patient outcomes and prevent future complications. |
| 45. | (Makhter, 2016)  Malaysia | Cross-sectional comparative study | 300  N WITH DFU=300 | 1.Socio- demographic characteristics data  2. Clinical characteristics data.  3. The short form McGill pain questionnaire (SF- MPQ).  4. The medical outcomes study 36-item short-form health survey (SF-36). - Diabetic foot ulcer scale- short form (DFS-SF).  5. Katz activities of daily living (Katz ADL). 6.Lawton instrumental activities of daily living (Lawton IADL). | To investigate the relationships between diabetic foot ulcer pain and  health-related quality of life and functional status in people over sixty years in  Malaysia. | All participants reported DFU pain, which was more severe in married females, those with multiple health issues, recurrent ulcers, Grade 4 ulcers, and those using antimicrobial dressings. Health-related quality of life and functional status declined with increasing pain. | Pain was negatively correlated with several HRQoL domains, including physical role, bodily pain, general health, vitality, social functioning, role emotional, and mental health. Scores for role physical, role emotional, and general health were notably low, reflecting significant impacts from diabetic foot ulcers (DFUs), while mental health and bodily pain scores were relatively higher, indicating some resilience. | Participants reported poor health-related quality of life (HRQoL) in physical functioning, physical role, and general health. Family support was found to enhance treatment adherence and emotional well-being, boosting patient confidence and motivation, although its direct effects on wound size and glycemic control were limited. The weak correlations between family support and HRQoL outcomes indicate that while family engagement is helpful, it is insufficient alone to mitigate the broader psychosocial burdens of DFU. | The paper does not examine if psychosocial factors influence the progression or recurrence of ulceration. Instead, it focuses on the relationship between pain, socio-demographic and clinical characteristics, and health-related quality of life in patients over sixty with diabetic foot ulcers. While it discusses how factors like pain correlate with reduced quality of life and functional status, it does not directly address psychosocial factors or their impact on the progression or recurrence of ulcers. The emphasis is primarily on clinical variables and their associations with pain and quality of life outcomes. |
| 46. | (Sigmon, 2021)  North Carolina, United States | Cross-sectional, descriptive, correlational research design | 64  N WITH DFU=64 | Data for the study were collected using an investigator developed demographic data form  and standardized instruments. The standardized instruments included: (a) Patient Activation Measure (PAM), (b) Family APGAR, (c) Patients’ Insights and Views of Teamwork Survey  (PIVOT), and (d) Short-Form 12 Version 2.0 (SF-12v2). | The purpose of this study was to examine the relationships between self-management behaviors (SM), family support (FS), patient perceptions of interprofessional collaborative teamwork (PP-IPCT), health-related quality of life (HRQoL), wound size (WS), self-monitored blood glucose (SMBG), and demographic factors in patients diagnosed with diabetic foot ulceration (DFU). The study sought to understand how these factors interact and influence each other in the context of managing DFU. | There is a weak correlation between IPC teamwork perceptions, family support, and self-management behaviors. Social facilitation (e.g., family support and IPC teamwork) indirectly influences self-management, improving glycemic control and wound management outcomes. | This paper outlines significant emotional consequences and psychosocial impacts of living with diabetic foot ulcers (DFU). Patients frequently experience emotional distress, including feelings of sadness, anxiety, and diminished confidence, which can hinder their ability to manage their condition effectively. Low scores in health-related quality of life (HRQoL) measures, particularly in role emotional and general health categories, indicate that DFU profoundly affects patients' daily activities and emotional well-being.  The findings highlight the importance of understanding patients' emotional states and the social dynamics that affect their health to develop more effective care strategies. | Weak correlations between family support and HRQoL outcomes indicate that while family engagement is helpful, it is insufficient alone to mitigate the broader psychosocial burdens of DFU. | Yes, the paper discusses the influence of psychosocial factors on the progression and recurrence of diabetic foot ulcers (DFU). It highlights that emotional distress, psychological well-being, and family support can significantly impact self-management behaviors, which in turn affect the likelihood of ulcer progression or recurrence. The findings suggest that addressing these psychosocial factors is crucial for improving patient outcomes and preventing future complications. |
| 47. | (Iverson et al. 2009)  Norway | Cross-sectional analysis | 65,126 i  N WITH DFU=155 | The study utilized several measures and instruments to assess psychological distress, perceived health, and well-being among participants:  Hospital Anxiety and Depression Scale (HADS):  Psychological Well-being Index:  Constructed from four self-assessed questions related to life satisfaction, vigor, calmness, and cheerfulness. Perceived Health Measure:  A single-item question asking participants to rate their health on a scale from 1 (poor) to 4 (very good). Demographic and Lifestyle Variables:  The study also collected data on demographic factors (age, gender, education), lifestyle factors (body mass index, smoking status), and cardiovascular conditions to control for these variables in the analyses. | The aim of the study was to compare levels of anxiety and depression, psychological well-being, and perceived health between three groups: individuals with diabetes who have a history of diabetic foot ulcers, individuals with diabetes without a history of foot ulcers, and individuals without diabetes. The study sought to investigate whether differences in these psychological outcomes could be explained by demographic characteristics, lifestyle factors, and cardiovascular conditions, in addition to diabetes-specific variables. Ultimately, the research aimed to enhance understanding of the emotional and psychosocial impacts of diabetic foot ulcers on individuals' health and well-being. | Individuals with diabetes and a history of DFU reported significantly poorer perceived health and psychological well-being compared to both non-diabetic individuals and diabetic individuals without foot ulcers. While depressive symptoms were notably higher in those with a history of foot ulcers, anxiety levels did not differ significantly between the groups. | The paper reveals that individuals with a history of DFUs experience significantly higher levels of depressive symptoms and poorer perceived health compared to both non-diabetic individuals and diabetic individuals without DFUs. While anxiety levels did not show significant differences among these groups, the study emphasizes that the psychological burden of having a DFU extends beyond physical health, affecting overall life satisfaction and emotional well-being. | Diabetic foot ulcers significantly impair perceived health and psychological well-being, reflecting the broader impact of this complication beyond its physical effects. •Higher levels of depression symptoms, though not statistically significant after adjustments, were notable in the DFU group. Depression often leads to social withdrawal, further exacerbating loneliness and reducing social networks. • Lower scores in life satisfaction and calmness suggest that individuals may struggle to maintain harmonious social relationships. This could stem from chronic worry about their condition or frustration with limitations imposed by their health. | The paper does not explicitly state that psychosocial factors influence the progression or recurrence of ulceration. However, it emphasizes the significant emotional and psychological challenges faced by individuals with a history of diabetic foot ulcers, suggesting that these psychosocial factors could potentially impact overall health management and, indirectly, the risk of ulcer recurrence. |
| 48. | (Setyawati & Sagita, 2024)  Indonesia | Clinical Case Study | 1  N WITH DFU=1 | The study utilized the Perceived Stress Scale (PSS) to assess the patient's stress levels, which is a 10-item questionnaire measuring perceived stress on a scale from 0 to 40. Additionally, psychological interventions included individual counseling sessions, cognitive behavioral techniques, and stress management strategies to address the emotional challenges faced by the patient. | The aim of the study was to showcase the psychological complexities encountered in managing diabetic foot ulcers (DFUs) and to highlight the importance of integrating psychological assessments and interventions into treatment protocols. It sought to demonstrate how targeted psychological support could positively influence emotional health and improve treatment adherence and wound healing outcomes in patients with DFUs. | Integrating psychological interventions significantly reduced the patient's perceived stress levels and improved emotional well-being, leading to better treatment adherence and enhanced wound healing outcomes. The results underscore the importance of addressing psychological factors in the management of DFU to optimize patient care and QoL. | •The patient experienced high levels of stress due to societal expectations, feelings of isolation, and the stigma associated with her condition. •Psychological distress manifested as frustration, fear, and decreased motivation to manage diabetes, compounding her health issues. | •Social stigma around being unmarried and diabetic led to emotional isolation and further exacerbated the psychological burden. •Preexisting stress negatively influenced the patient's self-care behaviors, including adherence to glucose monitoring and medical appointments. •Lack of consistent support from family and peers resulted in poor adherence to medical recommendations, impacting treatment outcomes. Involvement of family and support groups was crucial in addressing these psychosocial challenges. | Yes, the paper indicates that psychosocial factors significantly influence the progression and recurrence of ulceration. Psychological stress, societal stigma, and emotional distress are highlighted as barriers to treatment adherence, which can exacerbate the severity of diabetic foot ulcers and hinder healing. The study emphasizes that addressing these psychosocial factors is crucial for effective management and prevention of ulcer recurrence. |
| 49. | (Ismail et al. 2007) | Prospective cohort study | 253  N WITH DFU=253 | The study utilized several measures and instruments to assess various factors related to the participants:  Schedules for Clinical Assessment in Neuropsychiatry 2.1 (SCAN 2.1): This semistructured diagnostic interview was used to identify participants who met the DSM-IV criteria for major and minor depressive disorders.  Clinical Assessments: Baseline assessments included demographic information and potential covariates such as age, sex, marital status, socioeconomic status, smoking status, and alcohol use.  University of Texas Classification: This classification system was employed to categorize the severity and size of the ulcers.  A1C Measurements: A1C levels were measured at baseline and at 12 and 18 months to assess glycemic control.  Standardized Checklists: These were used by clinic podiatrists to record the status of ulcers, including healing, recurrence, and any amputations during the follow-up period. | The aim of the study was to evaluate whether depression was associated with mortality in individuals with their first diabetic foot ulcer over an 18-month period. | This study investigates the association between depression and mortality in individuals with their first diabetic foot ulcer (DFU) over 18 months. It finds that one-third of patients have clinical depression, which significantly increases the risk of mortality, while no significant link is found between depression and glycemic control. | This study reveals a high prevalence of depression among patients with diabetic foot ulcers, with 8.1% experiencing minor depression and 24.1% major depression, indicating that nearly one-third of participants suffer from clinically significant depression. This emotional burden is associated with a threefold increase in mortality risk over 18 months and includes fear of complications like amputation, anxiety, and social isolation, all of which diminish quality of life and reduce adherence to diabetes self-care practices. Individuals with major depressive disorder are less likely to engage in necessary self-care, worsening their condition. The fear of complications further exacerbates anxiety and distress, significantly impacting overall quality of life and contributing to feelings of hopelessness and despair. | The study highlights that the stigma surrounding visible disabilities, like foot ulcers, often leads to social isolation, as patients may feel embarrassed or anxious about their condition, resulting in reduced social interactions and support that worsen their emotional state. Furthermore, diabetic foot ulcers significantly impair not only physical health but also psychosocial well-being, as individuals face emotional distress and complications that diminish their overall quality of life while managing both their diabetes and the emotional repercussions of their condition. | This paper emphasizes the impact of depression on overall health and mortality. However, the relationship between psychosocial factors and ulcer recurrence is not explicitly addressed/it did not establish a direct link between mental health issues and the recurrence of foot ulcers. |
